# Multifunctional ACE2-nanobody fusion design for pan-specific neutralization and cardiovascular protection in SARS coronavirus infection

**DOI:** 10.1128/aac.00024-26

**Published:** 2026-03-23

**Authors:** Traian Sulea, Matthew Stuible, Maria Moreno, Alex Pelletier, Martin A. Rossotti, Yuneivy Cepero-Donates, Jacqueline Slinn, Patrick Salois, Anh Tran, Melissa Hewitt, Alvaro Yogi, Nazanin Rohani, Brian Cass, Anne E. G. Lenferink, Jamshid Tanha, Binbing Ling, Etienne Lessard, Laurence Delafosse, Jagdeep K. Sandhu, Danica Stanimirovic

**Affiliations:** 1 Human Health Therapeutics Research Centre, National Research Council Canada 729201 , Montreal, Quebec, Canada; 2 Institute of Parasitology, McGill University 242136, Sainte-Anne-de-Bellevue, Quebec, Canada; 3 Human Health Therapeutics Research Centre, National Research Council Canada, Ottawa, Ontario, Canada; 4 Department of Biochemistry, Microbiology and Immunology, University of Ottawa 151173, Ottawa, Ontario, Canada; 5 Center for Infection, Immunity and Inflammation, University of Ottawa 6363, Ottawa, Ontario, Canada; Chinese Academy of Medical Sciences and Peking Union Medical College, Beijing, China

**Keywords:** COVID-19, spike protein, virus neutralization, blood pressure reduction, ACE2 enzyme activity, single-domain antibody, albumin binding

## Abstract

Severe acute respiratory syndrome coronaviruses use the ACE2 receptor for viral entry while downregulating its activity, potentially leading to hypertension and major organ injuries. Dual-action technologies based on soluble ACE2 aimed to neutralize the virus while restoring ACE2’s normal enzymatic activity. Here, we describe a novel general molecular design, VHH_Spike_-ACE2_ECD_-VHH_Albumin_, in the toolbox of ACE2-centric therapeutic modalities. The optimized nanobody module VHH_Spike_ afforded strong pan-specific binding against the entire sarbecoviral clade. This correlated with potent *in vitro* neutralization of pseudotyped virus variants of concern, with IC_50_ values in the picomolar range. Exogenous enzymatic activity was provided by the ACE2_ECD_ module, which also contributed binding avidity via intrinsic homodimerization. Persistence of enzymatic activity in circulation was increased *in vivo* via the nanobody module VHH_Albumin_ optimized for serum albumin binding. Single-dose therapeutic administration of lead compound 72opt-ACE2-R28 demonstrated virus neutralization in lungs of hamsters at day 5 post-infection with SARS-CoV-2. In hypertensive mice maintained under continuous injection of angiotensin II, a single dose of 72opt-ACE2-R28 normalized systolic blood pressure, maintaining a 30 mmHg reduction after 24 h. Overall, encouraging coronavirus neutralization and hypertension reduction showed magnitudes and timeframes appropriate for treatment of typical acute infections. We discuss molecular bases of interactions with coronavirus spike protein molecules and future scale-up manufacturability toward clinical development of this modular design scaffold with high potential against emerging SARS-CoV-2 variants.

## INTRODUCTION

During severe acute respiratory syndrome (SARS) coronaviral infections, the human angiotensin-converting enzyme 2 (ACE2) receptor is used for viral entry into the host cell, leading to viral replication (1, 2). Additionally, SARS-CoV-2 infections downregulate ACE2 function via its ectodomain shedding, leading to accumulation of the vasoconstrictor angiotensin (Ang)-II and depletion of the protective Ang-(1–7), with potential to cause acute respiratory distress syndrome (ARDS), hypertension, acute kidney injury (AKI), and other major organ injuries (3–8). Hence, several dual-action technologies were developed that rely on soluble human ACE2 ectodomain (ACE2_ECD_) for trapping the virus and preventing it from entering and infecting host cells, as well as for providing an exogenous source of enzymatic activity for converting Ang-II to Ang-(1–7).

Starting with unmodified human ACE2_ECD_ as an initial therapeutic prototype (9, 10), several approaches for improvement have been explored. First, the ACE2_ECD_, despite being the natural viral entry receptor with wide cross-reactivity across virus variants of concern related to SARS-CoV-2, has a relatively weak binding affinity for the viral spike (S) protein compared to the affinities of clinical-grade antibodies (11, 12). Thus, human ACE2_ECD_ has been engineered through mutations that augment binding to the viral spike protein (10–12). However, these artificial mutations could increase the risk of immunogenicity, render the enzyme less active or inactive, and pose challenges for manufacturability due to decreased thermal stability, increase in aggregation propensity, and/or reduction of production yield, as observed for some of the affinity-improved mutants (11, 12). Second, the half-life of soluble ACE2_ECD_ in circulation is surprisingly low (13, 14), despite its relatively high molecular weight, which is well above the ~50 kDa threshold for rapid kidney excretion (15). Fusion of ACE2_ECD_ to the human IgG1 Fc domain was used as a classical method to prolong its half-life in circulation while additionally affording increased potency for virus neutralization via bivalency-induced binding avidity (16–18). However, interaction of the wild-type Fc fragment with Fc gamma receptors (FcγRs) on immune cells could potentially elicit adverse effects via an exaggerated immune response in COVID-19 (19). This prompted the design of direct fusions of ACE2_ECD_ to serum albumin or albumin-binding domains (ABD) to promote albumin-FcRn-mediated recycling (14, 20, 21).

The current study aims to explore novel protein engineering strategies within the ACE2-albumin paradigm by taking advantage of the favorable properties of single-domain antibodies (sdAbs, VHHs), such as superior stability, solubility, and modularity compared with other binding moieties. First, we designed ACE2-fusion constructs with increased S-protein binding affinity while maintaining broad cross-reactivity by largely deferring S-protein binding to a fused VHH module previously optimized for spike pan-specificity. For introducing binding avidity to membrane-anchored S proteins, instead of relying on the aforementioned Fc-fusing strategy or on other oligomerization domains (22), we used the full-length ACE2_ECD_ which is capable of non-covalent homodimerization via its Neck domain (2). Second, to maintain albumin-FcRn-based strategy for half-life extension, we fused an additional VHH module that binds serum albumin from multiple species, including human and rodents (23, 24). This approach substantially reduces the molecular weight relative to an albumin fusion and could offer a more readily tunable alternative to ABD fusions in terms of scalable biomanufacturing and reduced immunogenicity. Importantly, the required enzymatic activity of the ACE2 ectodomain was retained.

**Fig 1:**
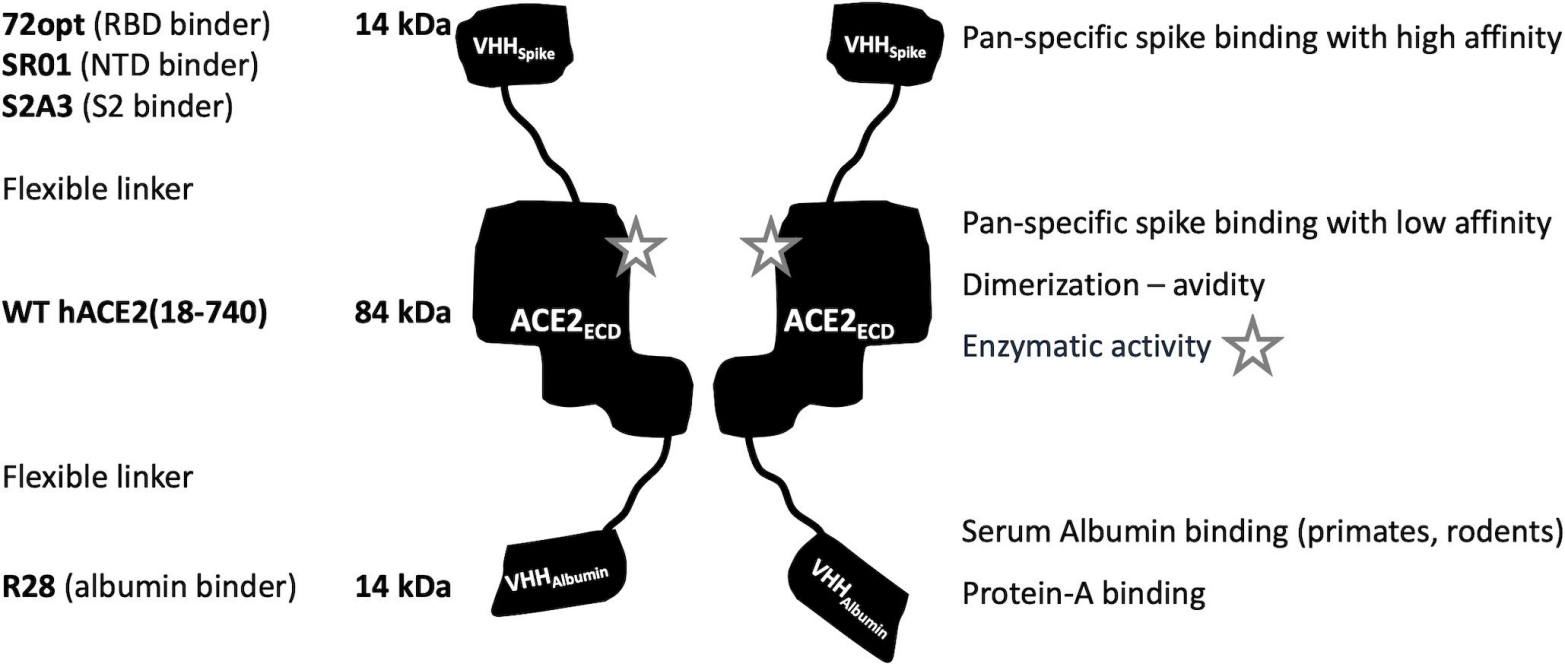
Design strategy of VHH_Spike_-ACE2_ECD_-VHH_Albumin_ fusions. Flexible linkers are used to fuse an S-protein binding module (VHH_Spike_) to the ectodomain of human ACE2 receptor (ACE2_ECD_) and then to a serum albumin binding module (VHH_Albumin_). The homodimeric assembly and enzymatic activity of the fusion construct are highlighted. Example modules are listed on the left, whereas key properties of the modules are listed on the right.

This novel design introduced here is depicted in Fig. 1 and follows a tripartite design, VHH_Spike_-ACE2_ECD_-VHH_Albumin_, where (i) VHH_Spike_ is a pan-specific high-affinity single-domain antibody targeting the viral S protein; (ii) ACE2_ECD_ is the full-length human wild-type ACE2 ectodomain, non-covalently dimerized and enzymatically active; and (iii) VHH_Albumin_ is a human serum albumin-binding single-domain antibody. This novel ACE2-based fusion design provides key advantages: (i) robust cross-reactivity against a wide spectrum of sarbecoviruses due to concerted action of VHH_Spike_ and ACE2_ECD_ modules; (ii) improved *in vivo* pharmacokinetics (PK) due to the VHH_Albumin_ module; (iii) potent *in vivo* virus neutralization due to high-affinity and neutralizing properties of the VHH_Spike_ module; and (iv) retention of ACE2 enzymatic activity, enabling mitigation of cardiovascular pathologies associated with viral infection.

In the current study, several examples of VHH_Spike_-ACE2_ECD_-VHH_Albumin_ variants were produced in mammalian cells and purified. The performance of this design was first validated *in vitro* by profiling pan-reactivity against a comprehensive panel of sarbecoviral S proteins, and then by assessing *in vitro* neutralization efficacy against a subset of S proteins from the main variants of concern. The lead VHH_Spike_-ACE2_ECD_-VHH_Albumin_ variant was further pursued through *in vivo* studies to demonstrate improved PK in blood, live virus neutralization, and stabilization of cardiovascular effects in normotensive and hypertensive animals. Data obtained here at the proof-of-concept level of this study demonstrate an overall encouraging performance of this novel ACE2-based scaffold, with additional refinements to be explored during a subsequent developmental stage.

## RESULTS

### Fusion design

Fusion constructs were designed according to the general formula VHH_Spike_-ACE2_ECD_-VHH_Albumin_. The central region consisted of the full ACE2 ectodomain sequence, to which different VHH domains were fused via flexible linkers at its N- and C-termini (Fig. 1; Table S1).

The ACE2_ECD_ was designed to include the entire ectodomain, consisting of residues 18–740, of the natural human receptor. This allows for non-covalent dimerization of the fusion construct via the Neck domain (residues 615–740), which follows the enzymatically active ACE2 catalytic domain (residues 18–614) (2). The ability of the designed fusions to homodimerize was deemed important to improve binding avidity toward the target S trimer; in the absence of other dimerization domains, for example, the IgG-Fc fragment, inclusion of the ACE2 Neck domain was therefore considered as a key feature of the present design. In addition to dimerization, the ACE2_ECD_ module was designed to retain enzymatic activity to mitigate ARDS and other organ injuries ensued by downregulation of the natural ACE2 receptor during SARS-CoV-2 infection (3, 4). As the natural receptor for viral entry into the host cell, the ACE2_ECD_ module exhibits exquisite cross-reactivity against S proteins across the SARS-CoV clade. However, it exhibits relatively low binding affinity toward the S protein, with equilibrium dissociation constants in the range of 6 nM–25 nM for monovalent interactions with various virus variants (25). In addition, the geometry of the ACE2_ECD_ homodimer is incompatible with both monomers binding simultaneously to a given S trimer (2) (Fig. S1). Both these characteristics of the ACE2_ECD_ were considered in the design of the dimeric VHH_Spike_-ACE2_ECD_-VHH_Albumin_ fusion constructs in order to defer bivalent binding to the S trimer primarily to the two VHH_Spike_ domains, with minimal contribution from the ACE2_ECD_ domains to S-protein binding.

**Fig 2:**
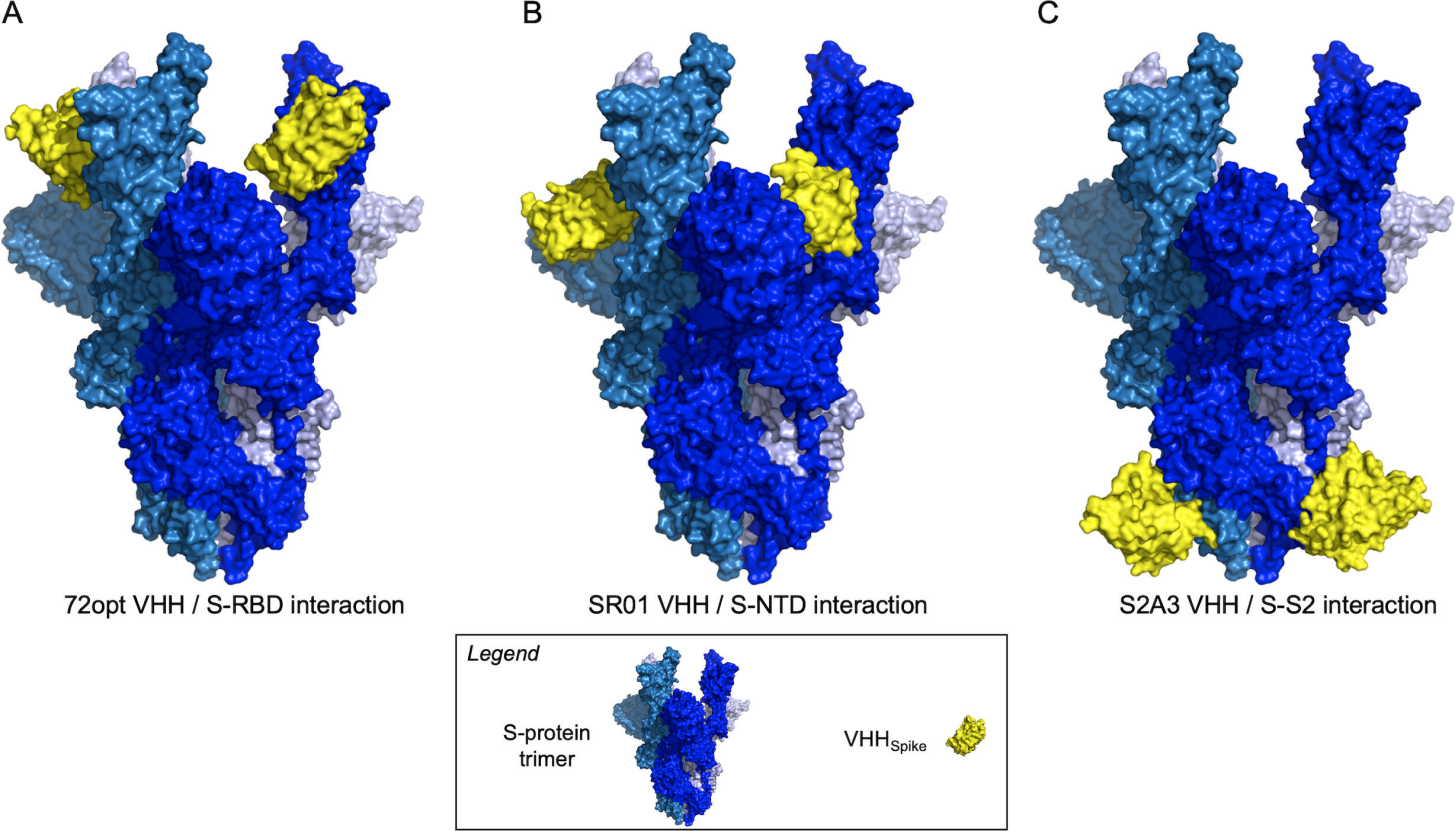
Binding modes of VHH_Spike_ modules to the S-protein trimer. The S trimer is shown in the 3-RBD-up conformation (PDB entry 7A98) in tones of blue. Two VHH_Spike_ modules bound to the S trimer are shown in yellow. (**A**) Predicted complex of the S trimer with the RBD-binder VHH 72opt. The binding mode is inferred from the crystal structure of the RBD-bound VHH72 (PDB entry 6WAQ). (**B**) Predicted complex of the S trimer with the NTD-binder VHH SR01. The binding mode was predicted with AF3 and confirmed by HDX-MS (Fig. S2A) (26). (**C**) Predicted complex of the S trimer with the S2-binder VHH S2A3. The binding mode was predicted with AF3 and confirmed by HDX-MS (Fig. S2B) (26).

In this study, for the VHH_Spike_ module, we selected three sdAbs named 72opt, SR01, and S2A3, which were previously validated to possess the critical attributes of high affinity, cross-reactivity, and neutralization potency (26, 27). Moreover, these selections were found suitable for sampling binding interactions against different regions of the S protein, including the receptor binding domain (RBD) in the case of 72opt, the N-terminal binding domain (NTD) for SR01, and the S2 region for S2A3 (26, 27). Predicted binding modes to the S-protein trimer of these VHH_Spike_ are shown in Fig. 2. In the case of the 72opt RBD binder, its binding mode is inferred from the crystal structure of the parental VHH-72 antibody bound to the RBD (28). The binding modes of the SR01 NTD binder and S2A3 S2 binder were predicted with the AlphaFold3 (AF3) method (29), and were found to agree with the targeted epitopes previously determined by HDX-MS experiments (Fig. S2) (26). As these selected examples of VHH_Spike_ sdAbs were previously shown to retain binding affinity when positioned at the N-terminus of a human IgG Fc domain, and in the absence of other data informing binding dependence on domain positioning, we elected to position the VHH_Spike_ module at the N-terminus of the designed ACE2 fusions.

The VHH_Albumin_ module was chosen as an sdAb capable of binding to human serum albumin. The intended role for VHH_Albumin_ was to improve persistence of the designed construct in circulation, mediated by binding to the host albumin, which has long circulation half-life via the FcRn-mediated recycling mechanism (30). We selected R28, an anti-human albumin sdAb (23, 24), which displays good binding affinity and cross-reactivity against serum albumins from several rodent species, allowing its PK testing across animal models. In addition, R28 can bind to Protein A (as do 72opt and SR01 employed here as the VHH_Spike_ module), potentially allowing scaled-up biomanufacturing by standard affinity purification workflows. Previous studies indicated that R28 and other albumin-binding sdAbs are able to significantly increase blood circulation half-lives of various biologics when fused at their C-termini (23). Therefore, we positioned the VHH_Albumin_ module at the C-terminus of the constructs designed here.

Flexible Gly-Ser linkers were designed to connect the various modules of the construct (Table S1). To this end, the linker length between the VHH_Spike_ and ACE2_ECD_ modules was varied depending on the location of their respective binding epitopes on the S trimer. Using the structural data of the VHH-72 sdAb bound to the S-RBD epitope (28), a (G_4_S)_5_ linker was determined by molecular modeling to generously allow unhindered simultaneous binding of the two 72opt VHH_Spike_ modules in the homodimeric construct to two RBDs on the S-protein trimer (Fig. 2A). To reduce the possibility of O-glycosylation and allow full flexibility at the N-terminus of the fused ACE2_ECD_ module, the last Ser residue of the linker was eliminated, resulting in a 24-residue flexible linker (G_4_S)_4_G_4_. A longer, 29-residue (G_4_S)_5_G_4_ linker was used to fuse the NTD-binding VHH_Spike_ SR01 and ACE2_ECD_, given that the NTDs are positioned more centrally than the RBDs, which are more peripherally positioned on the S-protein trimer (Fig. 2B), especially when the RBDs adopt the “up” conformation (31). The epitope of the NTD-binding VHH_Spike_ module SR01 was mapped previously by HDX-MS (26). Models of the SR01 sdAb bound to the NTD, which agree with the HDX-MS-identified epitopes, indicate that the (G_4_S)_5_G_4_ linkers provide sufficient flexibility for simultaneous binding of the sdAb to the trimeric S protein (Fig. 2B). Finally, in the case of the construct incorporating the VHH_Spike_ sdAb S2A3 binding to the S2 region, the epitopes mapped by HDX-MS (26) and confirmed by AF3 are closer to each other on the S-protein trimer. This allows the simultaneous binding of the two VHH_Spike_ modules of the homodimeric fusion on the S-protein trimer using the 24-residue linker (G_4_S)_4_G_4_ (Fig. 2C). All these bivalent binding modes, which are mediated entirely by the two VHH_Spike_ modules, are incompatible with the 3rd RBD of the trimer being occupied by one of the ACE2_ECD_ modules in the dimeric fusion construct with the current linkers (Table S2). Trivalent binding would require overly long linkers; therefore, this option was excluded from the designs. It is plausible, however, that, depending on the density of S proteins on the viral membrane, the designed flexible linkers might allow binding of a single homodimer construct across two or more S trimers embedded within the virus surface. This could induce even stronger avidity effects, potentially increasing the capacity for virus neutralization. In all the constructs, the (G_4_S)_5_ flexible linker was used between the ACE2_ECD_ and VHH_Albumin_ modules, based on past experience with R28 in extending the half-life of biologics *in vivo* (23).

### Expression, purification, and characterization of designed fusions

**Fig 3:**
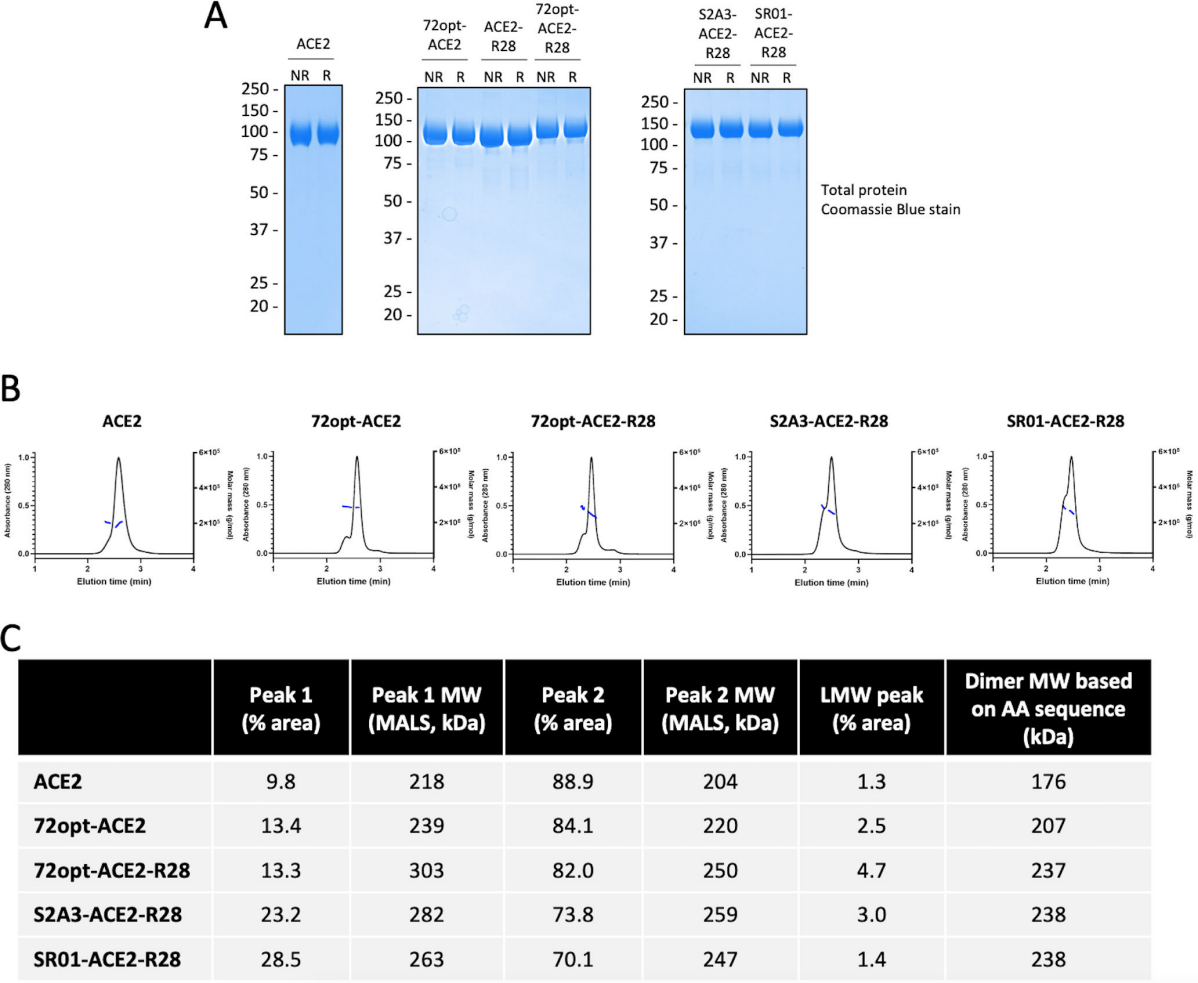
Production and purification of designed fusions. Fusion proteins were purified from CHO cell supernatants by IMAC, followed by Strep-Tactin affinity chromatography. Purified products were analyzed by SDS-PAGE, followed by total protein staining (**A**) and UPLC-SEC/MALS (**B**). Proteins analyzed in the three SDS-PAGE gels under reducing (R) and non-reducing (NR) conditions shown in **A** were from separate production runs at 100 mL scale. Molecular weight markers (in kDa) are indicated on the left of each gel panel. For UPLC-SEC/MALS results in **B**, the UV absorbance (280 nm) of the column eluate is shown in black, and the MALS molar mass estimates for the principal elution peaks are shown in blue. The estimated peak area percentages (based on UV absorbance signal) for the major peaks, as well as for minor low-molecular-weight (LMW) peaks, are tabulated in **C**, along with MALS molar mass estimates for the major peaks (average molar mass across each peak).

Three designed VHH_Spike_-ACE2_ECD_-VHH_Albumin_ fusions (72opt-ACE2-R28, S2A3-ACE2-R28, and SR01-ACE2-R28) and controls, including single-VHH fusions VHH_Spike_-ACE2_ECD_ (72opt-ACE2) and ACE2_ECD_-VHH_Albumin_ (ACE2-R28), and ACE2_ECD_ alone, were produced in the CHO^55E1^ cell line at 100 mL and 500 mL scales by transient expression. Yields (determined by a Protein A column/HPLC-based method at 7 days post-transfection) ranged between 100 and 350 mg/L in harvested culture supernatants. Secreted recombinant proteins were purified by a two-step purification process consisting of immobilized metal-ion affinity chromatography (IMAC) followed by Strep-Tactin chromatography, giving high-purity products (Fig. 3A). The fusion proteins were purified mainly as dimers, as expected, with the main component representing greater than 70% of the total protein (Peak 2, Fig. 3B and C). The secondary component is an undefined species of similar molecular weight based on multi-angle light scattering (MALS) (Peak 1, Fig. 3C), so it is unlikely to represent aggregates or multimers. To determine whether differential glycosylation could be responsible for Peak 1, purified proteins were treated with PNGase F (to hydrolyze N-linked glycans) and analyzed by SDS-PAGE and UPLC-SEC alongside untreated proteins. As shown in Fig. S3A, PNGase F treatment resulted in a small increase in mobility by SDS-PAGE, consistent with glycan removal; however, there was no decrease in the abundance of Peak 1 relative to Peak 2 (Fig. S3B; Table S3), indicating that Peak 1 is not simply a more highly glycosylated form of Peak 2.

### Binding cross-specificity

**Fig 4:**
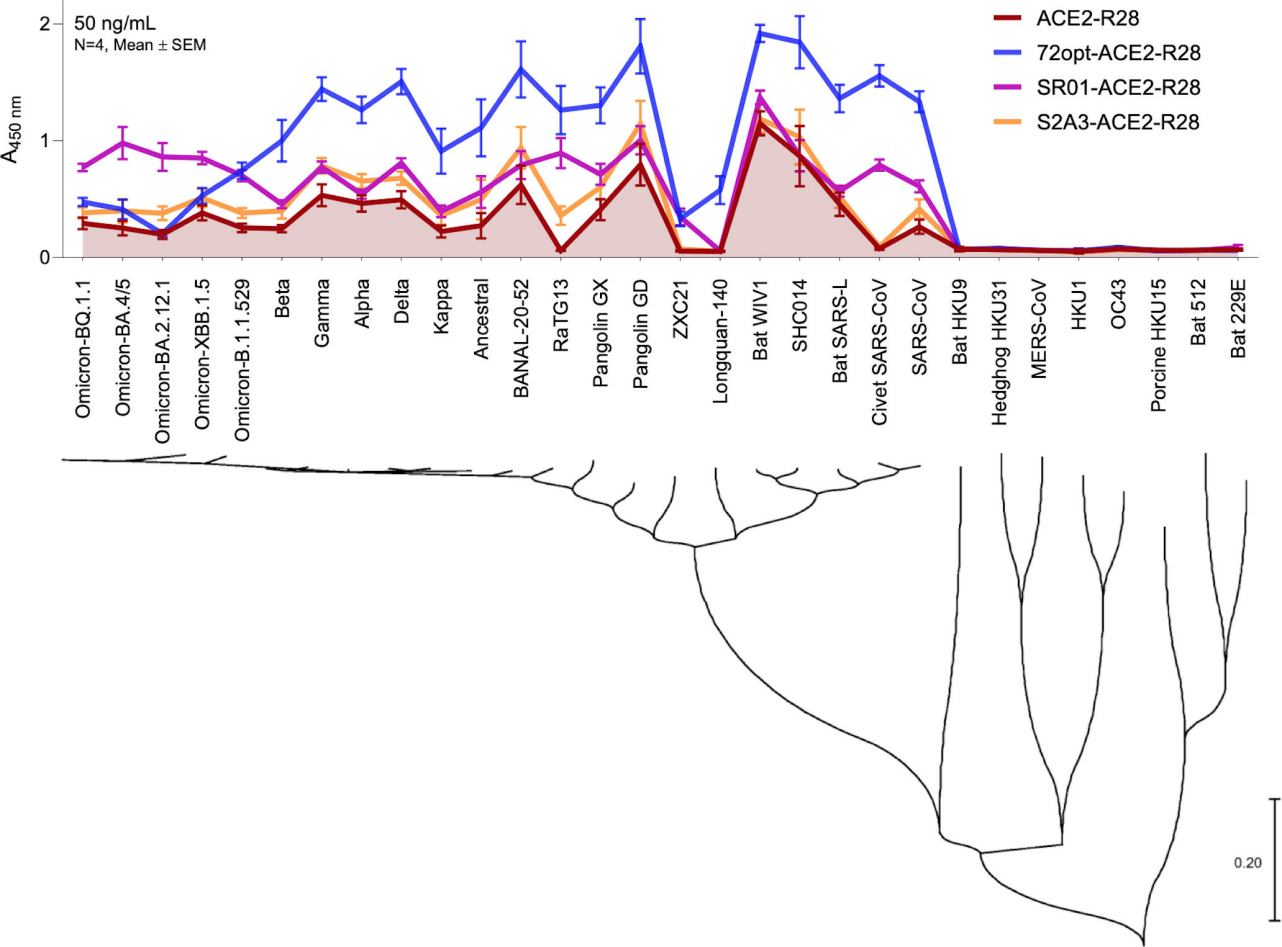
Cross-reactivity of the VHH_Spike_-ACE2_ECD_-VHH_Albumin_ fusions against recombinant S proteins from a panel of sarbecoviruses and other potentially zoonotic coronaviruses. ELISA was performed at a single concentration of VHH_Spike_-ACE2_ECD_-VHH_Albumin_ (50 ng/mL), and binding of the fusion constructs to S proteins was detected with anti-VHH:horseradish peroxidase. ACE2-R28 was included as a reference to highlight the broader cross-reactivity of the VHH_Spike_-ACE2_ECD_-VHH_Albumin_ constructs, which incorporate various VHH_Spike_ (72opt, SR01, or S2A3) targeting different regions of the spike protein (RBD, NTD, and S2, respectively). The phylogenetic tree of spike glycoproteins was constructed using MEGA11 (32).

The binding profile of the three VHH_Spike_-ACE2_ECD_-VHH_Albumin_ fusions (72opt-ACE2-R28, S2A3-ACE2-R28, and SR01-ACE2-R28) and the control ACE2-R28 was assessed against a large panel of recombinantly produced S proteins from 30 coronavirus variants covering SARS-CoV-2 and SARS-CoV clades, as well as more distant coronaviruses of human and animal origins (Fig. S4; Table S4) (26). As shown in Fig. 4 and Fig. S5, all three fusions had a broader cross-reactivity binding profile relative to the control ACE2_ECD_-VHH_Albumin_ fusion ACE2-R28, indicating the beneficial role of combining the ACE2_ECD_ and a VHH against the S protein. Reactivity was observed against human and animal virus variants from the clades of SARS-CoV-2 (16 variants) and SARS-CoV (6 variants) but did not extend to the more distant coronaviruses tested in this study (8 variants).

Among the panel of 22 S proteins tested from SARS-CoV and SARS-CoV-2 clades, the 72opt-ACE2-R28 fusion incorporating the RBD-binding sdAb was the broadest binder against 16 S proteins, whereas the SR01-ACE2-R28 fusion incorporating the NTD-binding sdAb was the broadest binder against four Omicron S proteins, with the two fusions having similar binding capacities for the remaining two S proteins. The S2A3-ACE2-R28 fusion incorporating the S2-specific sdAb was not found to be the best binder against any of them but was still superior to the reference ACE2-R28 fusion on most S proteins. In fact, the control ACE2-R28 fusion was the poorest binder on all S proteins tested. Encouragingly, VHH_Spike_-ACE2_ECD_-VHH_Albumin_ fusions conferred binding to S proteins of four virus variants with undetected binding for the control ACE2-R28 fusion: RaTG13, ZXC21, Longquan-140, and civet SARS-CoV; particularly, the 72opt-ACE2-R28 fusion had high binding to two of them and moderate binding to the remaining two. Overall, the combination of 72opt-ACE2-R28 and SR01-ACE2-R28 covers the entire spectrum of tested human and animal variants from the SARS-CoV and SARS-CoV-2 sarbecovirus clades, with medium to very high binding reactivity.

### Pseudovirus neutralization

**Fig 5:**
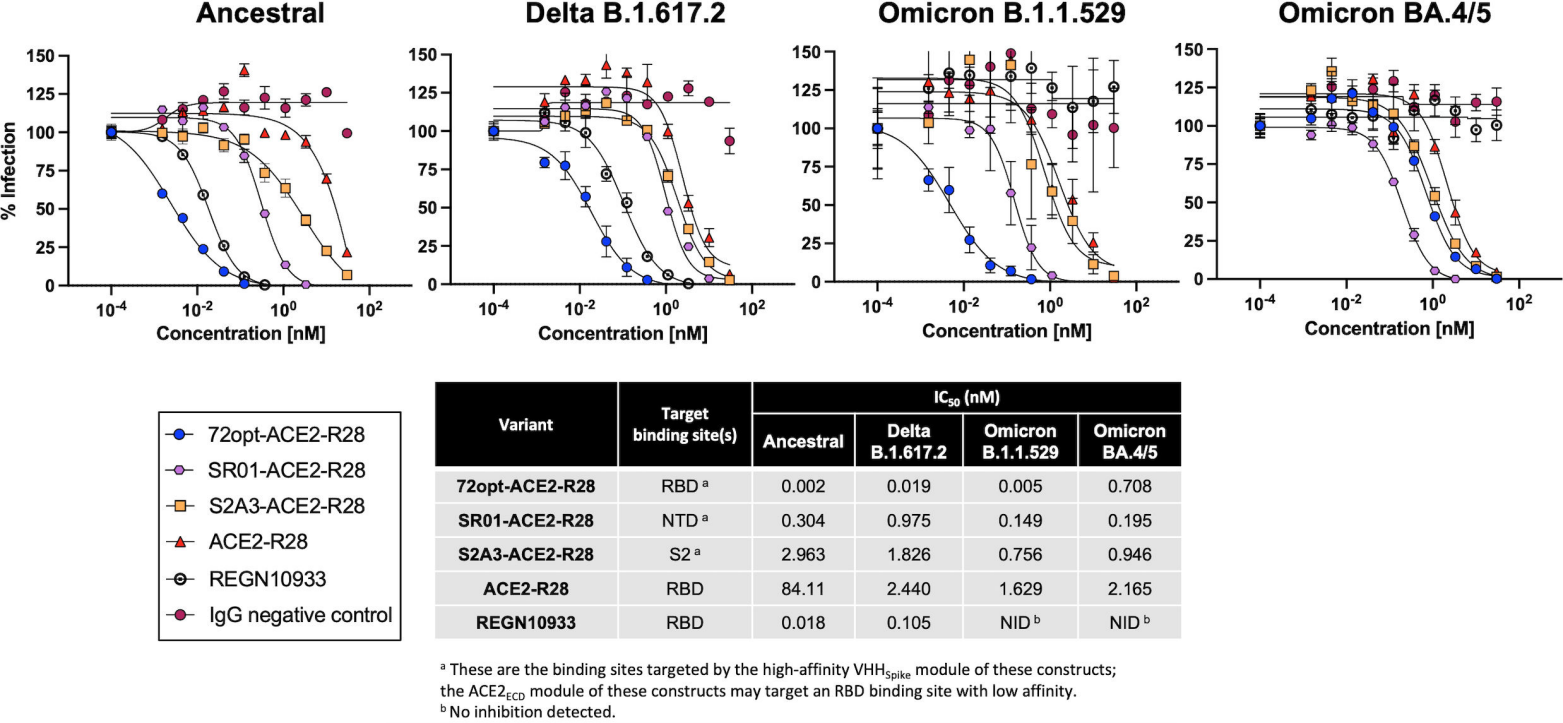
*In vitro* neutralization potency of VHH_Spike_-ACE2_ECD_-VHH_Albumin_ fusions against representative pseudotyped SARS-CoV-2 variants. The three VHH_Spike_-ACE2_ECD_-VHH_Albumin_ fusion constructs were titrated on HEK293T cells overexpressing human ACE2/TMPRSS2 to assess their neutralization potencies against ancestral SARS-CoV-2 and variants Delta B.1.617.2, Omicron B.1.1.529, and Omicron BA.4/5. In the assay, the ACE2-R28 fusion protein served as a reference, the REGN10933 (casirivimab) monoclonal antibody (34) acted as a positive control with known neutralization capacity, and a human IgG isotype was included as a negative control. The percentage of infection was determined by luciferase readout. Reported IC_50_ values were calculated from best-fit curves to data from duplicate experiments.

All three fusions were then tested for their ability to neutralize the infectivity of pseudotyped virus variants on human HEK293T cells recombinantly over-expressing human ACE2 and TMPRSS2 receptors (33). The *in vitro* cell-based neutralization assay was employed for the fusion constructs to analyze the neutralization potency against four SARS-CoV-2 variants from the aforementioned panels: the ancestral strain (i.e., the original Wuhan variant), the Delta variant of concern, and two of the Omicron variants, which were the most recent at the time of the study (Fig. 5). A positive control antibody REGN10933 (casirivimab) (34) and a negative control human IgG antibody were also included. In agreement with the binding cross-reactivity data (Fig. 4), the 72opt-ACE2-R28 fusion incorporating the RBD-specific sdAb was the most potent neutralizer against the ancestral, Delta, and Omicron B.1.1.529 variants, with IC_50_ values of 2 pM, 19 pM, and 5 pM, respectively. The SR01-ACE2-R28 fusion incorporating the NTD-binding sdAb was the most potent binder against the Omicron BA.4/5 variant, with an IC_50_ in the sub-nanomolar range (0.195 nM). The S2A3-ACE2-R28 fusion incorporating the S2 region-binding sdAb was the weakest neutralizer for all four virus variants. All three fusions were more potent neutralizers than the reference ACE2-R28 lacking the VHH_Spike_ module (Fig. 5), consistent with the binding cross-reactivity data (Fig. 4). Relative to the REGN10933 (casirivimab) benchmark antibody, the 72opt-ACE2-R28 fusion was significantly more potent against the ancestral and Delta variants and retained binding and neutralization to Omicron variants for which neutralization could not be detected with casirivimab.

### Pharmacokinetics

**Fig 6:**
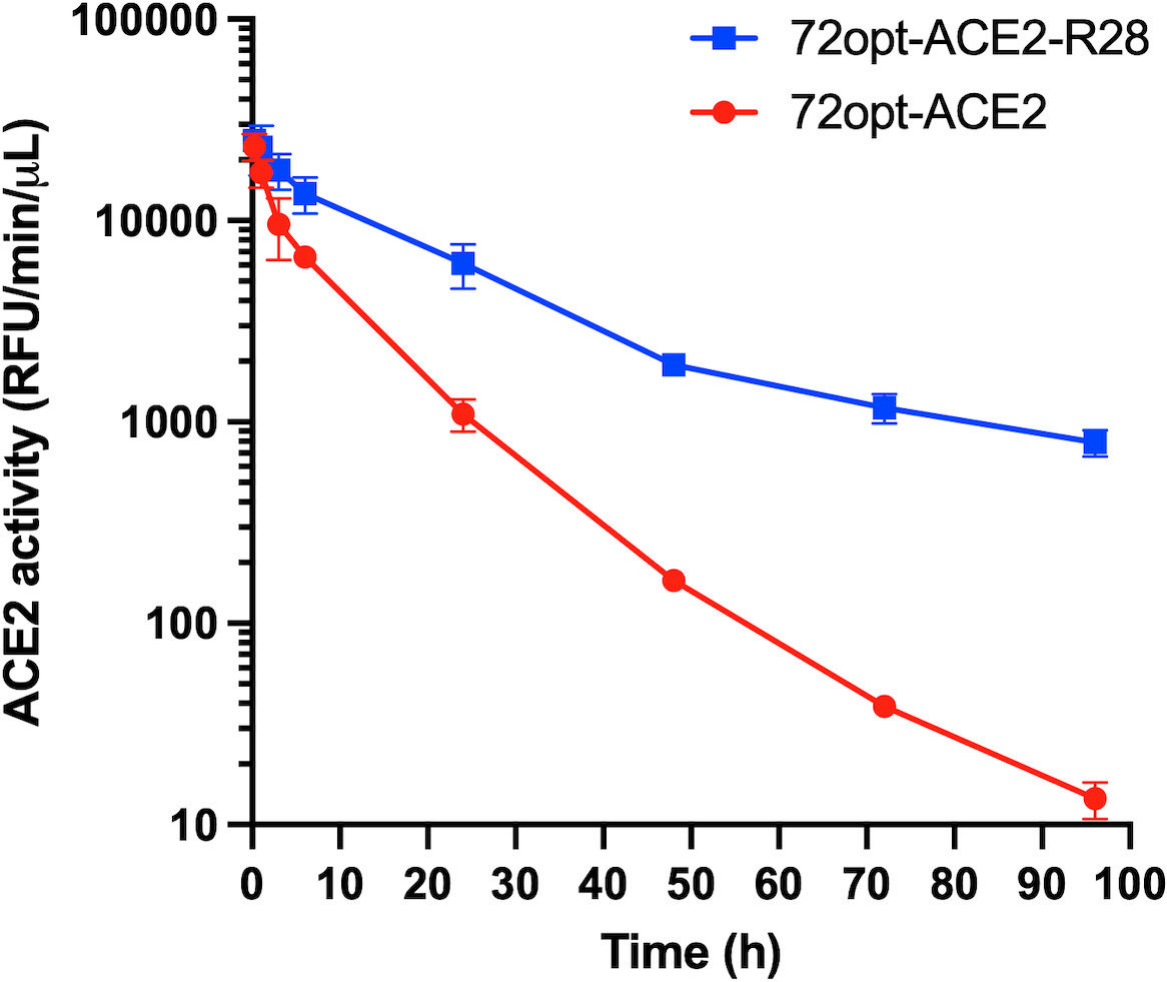
Pharmacokinetics of the lead fusion 72opt-ACE2-R28. Serum ACE2 enzymatic activity over time (mean ± SD; *n* = 2), following IV administration of 10 mg/kg of either 72opt-ACE2 or 72opt-ACE2-R28 diluted in PBS to naïve male Wistar rats. RFU, relative fluorescence unit.

Before testing virus neutralization in animal models, we examined the PK of the fusion constructs *in vivo* in order to verify the half-life extension conferred by their VHH_Albumin_ module. To this end, we compared two constructs, the lead candidate 72opt-ACE2-R28, which includes the albumin-binding R28 module, and 72opt-ACE2, the control construct lacking R28. Figure 6 presents the PK profiles of these molecules in the blood of healthy rats. The PK behavior was monitored via the enzymatic activity of the ACE2 domain in collected serum samples. Following a single intravenous (IV) administration, 72opt-ACE2-R28 shows a 2.8-fold higher serum exposure (area under the curve; *AUC_0-96h_*) compared to 72opt-ACE2 (444 vs 157 h * RFU/min/mL), which can be attributed to the VHH_Albumin_ module.

### *In vivo* virus neutralization efficacy

**Fig 7:**
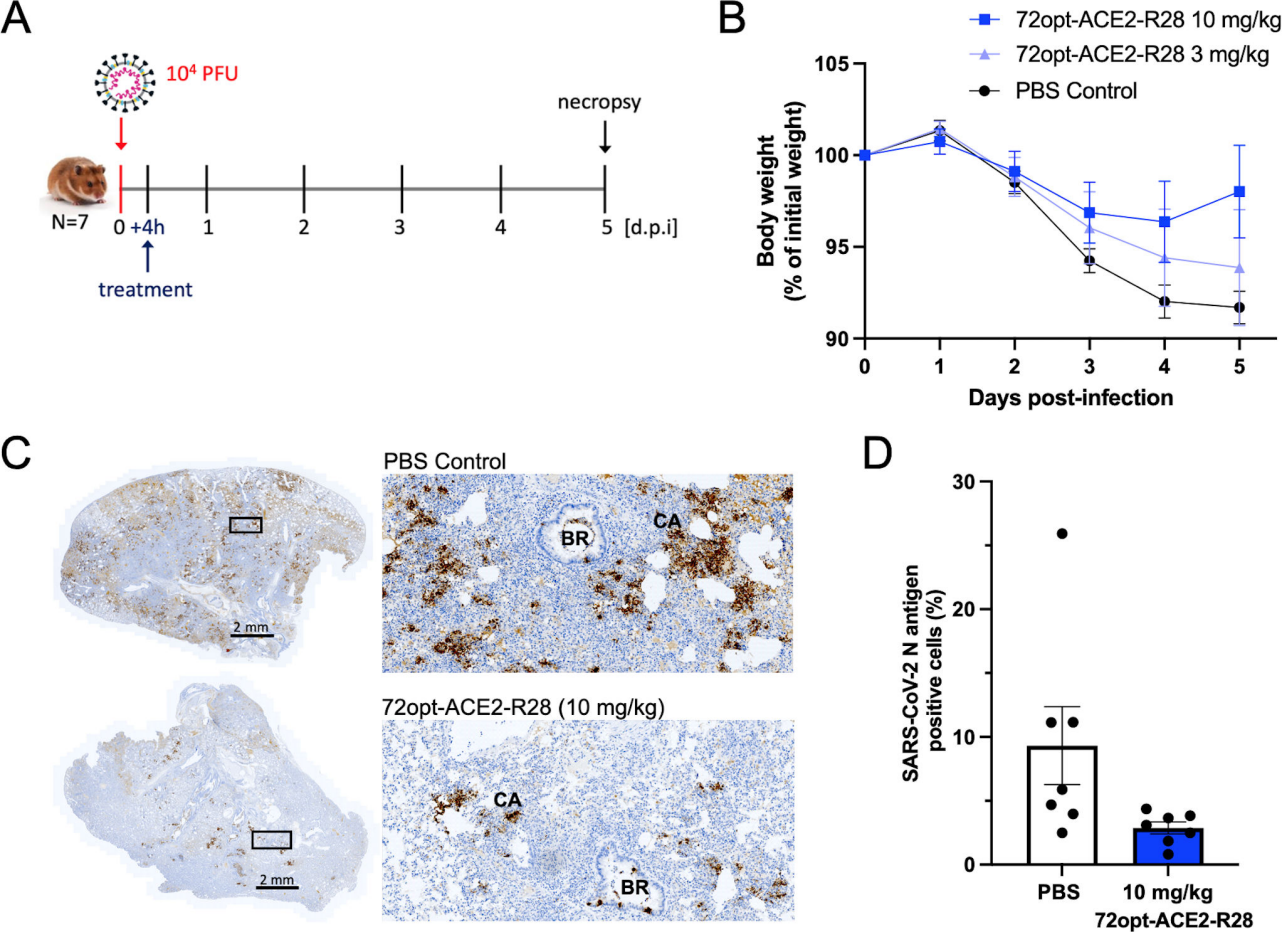
*In vivo* virus neutralization by the lead construct 72opt-ACE2-R28. (**A**) Male hamsters were challenged intranasally with 10^4^ plaque-forming units (PFUs) of SARS-CoV-2 ancestral isolate. Four hours later, they were administered intravenously via the retro-orbital vein with 150 µL of 72opt-ACE2-R28 fusion at a single dose of either 3 mg/kg or 10 mg/kg. Each group consisted of 7 animals. [d.p.i.], days post-infection. (**B**) Body weight measurements during the time course of the experiment (mean ± SEM). (**C**) Immunohistochemical (IHC) detection of SARS-CoV-2 N antigen in the lungs of infected animals necropsied at day 5 post-infection. Lung tissues from infected, untreated PBS control animals and from animals treated with 10 mg/kg of 72opt-ACE2-R28 were subjected to IHC and probed using anti-SARS-CoV-2 N protein antibody, as described in the Materials and Methods section. Representative photomicrographs of nucleocapsid protein expression (dark brown) are shown. The N protein was localized mainly to macrophages in the CA, bronchiolar epithelium (BR), and exudate within the lumen of BR. A visible reduction in brown staining is observed after treatment with 10 mg/kg of 72opt-ACE2-R28. (**D**) Quantitative analysis of N protein staining on whole lung scans. Images were quantified using QuPath 0.3.2. Data shown are mean ± SEM from seven animals in each group. Statistical comparison between the two groups gave a two-sided *P*-value of 0.0175 based on a non-parametric Mann-Whitney U-test performed in Prism 10.

Upon demonstration of the half-life extension in rats, the lead fusion construct 72opt-ACE2-R28 was tested for viral neutralization in the hamster model of SARS-CoV-2 infection. The construct was delivered intravenously via the retro-orbital vein in a single therapeutic dose administered 4 h post-challenge with the ancestral SARS-CoV-2 strain (Fig. 7A). Two single doses were tested: 3 mg/kg and 10 mg/kg. In terms of body weight changes, the lower dose attenuated the weight loss relative to untreated infected animals, whereas the 10 mg/kg dose completely stopped the weight loss by day 4 post-infection and allowed for weight gain by day 5 (Fig. 7B). The effect on body weight at the higher dose was followed by an analysis of viral load reduction in the lungs of infected animals necropsied on day 5 post-infection (Fig. 7C). In the untreated infected animals (PBS control group), abundant expression of the SARS-CoV-2 nucleocapsid (N) protein was observed in large, multifocal consolidated areas (CA) throughout the lung parenchyma (Fig. 7C, top panels). Strong immunoreactivity was also detected in cellular exudates within the pulmonary bronchi (BR). In contrast, lung sections from animals treated with 10 mg/kg of 72opt-ACE2-R28 showed a marked reduction in N-antigen staining, with pronounced viral clearance observed (Fig. 7C, bottom panels). Quantification of images showed a significant reduction in N-antigen immunoreactivity in the group of animals treated with 72opt-ACE2-R28 relative to the untreated infected group (Fig. 7D). The reduction in tissue viral-antigen expression upon treatment with 72opt-ACE2-R28 is consistent with the strong pseudovirus neutralization by 72opt-ACE2-R28 (Fig. 5).

### *In vivo* blood pressure effect and general toxicity

SARS-CoV-2 infection triggers a local downregulation of the ACE2 receptor, disrupting the balance between the vasoconstrictor Ang-II and vasodilator Ang-(1–7), which causes an increase in blood pressure, as well as inflammation and glomerulosclerosis, leading to the progression of chronic kidney disease (3, 4). We thus investigated the protective effect of the lead fusion 72opt-ACE2-R28, which was enzymatically active *in vivo* (Fig. 6), on blood pressure. Two sets of *in vivo* experiments were conducted to assess its efficacy: one in normotensive mice and another in hypertensive mice (Fig. S6).

**Fig 8:**
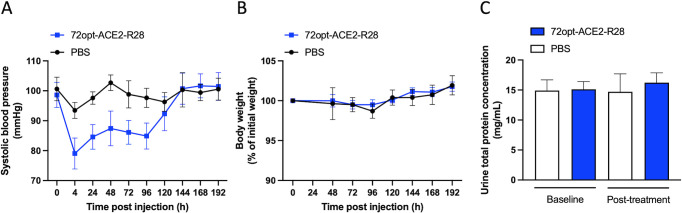
Cardiovascular effects of the lead construct 72opt-ACE2-R28 in normotensive animals. Male CD-1 mice were injected intravenously via the tail vein with 72opt-ACE2-R28 at a single dose of 10 mg/kg or PBS as vehicle control. Each group consisted of seven animals (mean ± SEM). (**A**) Systolic blood pressure was measured at the tail using a high-throughput blood pressure apparatus at predetermined time intervals post-injection. (**B**) Body weights were monitored daily and compared to the initial body weights of the animals at the time of injection. (**C**) Total protein concentration in urine samples was measured 7 days post-injection.

In normotensive mice, a single IV dose of 10 mg/kg 72opt-ACE2-R28 administered after a 3 week acclimatization led to a 15 mmHg reduction in systolic blood pressure (SBP) relative to untreated animals. This effect was sustained for 4 days, after which the SBP of treated animals gradually returned to a normal level (Fig. 8A). No significant changes in body weight (Fig. 8B) and total protein in urine (Fig. 8C) were observed during the course of the experiment for treated versus untreated groups of animals, which can be taken as preliminary indications of safety at the administered dose.

**Fig 9:**
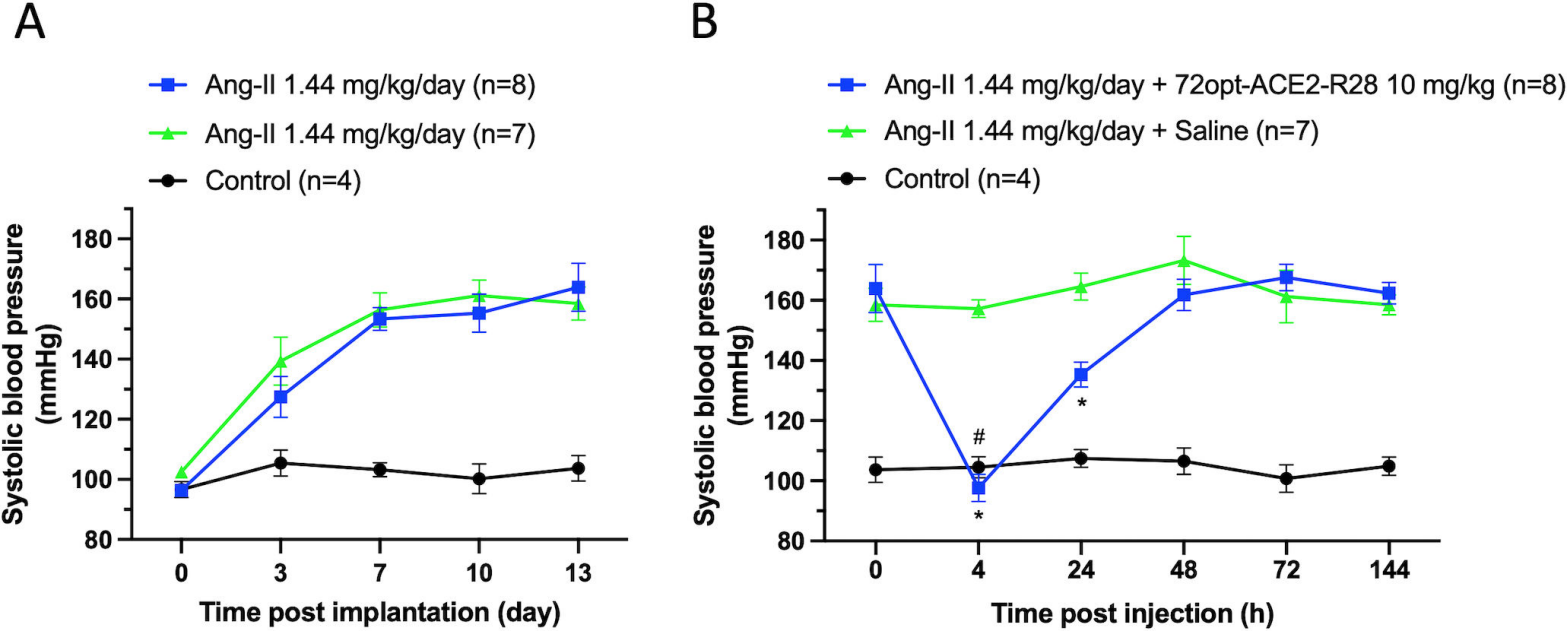
Cardiovascular effects of lead construct 72opt-ACE2-R28 in hypertensive animals. (**A**) Progression of systolic blood pressure over the 2 weeks of establishing hypertensive male CD-1 mice via implanted mini-pumps used to inject Ang-II daily at a rate of 1.44 mg/kg/day. Animals were split into two groups (*n* = 8 and *n* = 7) for follow-up administration of test articles. Measurements were taken every 3 days (mean ± SEM). The control group included four animals without the Ang-II mini-pump. (**B**) Effect of 72opt-ACE2-R28 on decreasing systolic blood pressure in established hypertensive mice. On the 14th day after the beginning of daily Ang-II infusion, 72opt-ACE2-R28 was injected at a single dose of 10 mg/kg into the tail vein of one hypertensive group (*n* = 8), while the other hypertensive group (*n* = 7) received IV saline injections. Daily Ang-II infusion continued at the same rate of 1.44 mg/kg/day during treatment. SBP was measured at the tail using the high-throughput blood pressure apparatus at predetermined time intervals post-injection. Statistical analysis of SBP reduction gave a *P*-value <0.005 (#) at 4 h versus 0 h in the treated group, and *P*-values <0.05 (*) at 4 h and 24 h in the treated versus saline groups, based on a two-way ANOVA test performed in Prism 10.

The Ang II-induced hypertensive mouse model was then used to simulate a cardiovascular disease scenario similar to that induced during SARS-CoV-2 infection. After an initial 2 weeks of acclimatization, animals were subcutaneously implanted with an osmotic pump to continuously infuse Ang-II at a rate of 1.44 mg/kg/day for an additional 2 weeks. As shown in Fig. 9A, by the end of the 2 week Ang-II infusion period, the SBP stabilized at 160 mmHg in the Ang-II-treated, that is, 60 mmHg higher than the SBP of the normotensive group. On this hypertensive background established after 14 days of Ang II infusion, animals were administered a single IV dose of either 10 mg/kg 72opt-ACE2-R28 or saline as a negative control. Treatment with the 72opt-ACE2-R28 fusion had a marked effect, reducing SBP by 60 mmHg within 4 h post-administration, effectively restoring SBP to the normotensive level (Fig. 9B). The effect remained statistically significant 24 h post-treatment, with SBP reduced by approximately 30 mmHg. However, the SBP returned to the initial hypertensive (untreated) level 2 days after treatment. It is important to note that the Ang II infusion continued at a constant rate throughout the study. This explains the shorter duration of the treatment effect in the hypertensive model (Fig. 9B) compared to the normotensive group (Fig. 8A). These findings suggest that daily administration of the 72opt-ACE2-R28 compound may be required to maintain the SBP at normotensive levels during prolonged hypertensive states, for example, in those that occur in SARS-CoV-2 infection. Another important note is that the magnitude of the SBP reduction elicited by a single 10 mg/kg dose was greater in hypertensive (60 mmHg drop) than in normotensive animals (15 mmHg drop), demonstrating a desired pharmacological behavior.

## DISCUSSION

This study explores novel multifunctional molecular designs, VHH_Spike_-ACE2_ECD_-VHH_Albumin_, centered around the ectodomain of human ACE2 receptor, the host cell entry point of SARS-CoV-2 and related sarbecoviruses. The two main attributes of ACE2 in anti-viral therapy are its inherent pan-reactivity against variants and enzymatic activity to convert angiotensin-II. The first attribute is required to ensure relevance against emerging virus variants, while the second one is beneficial to counteract ACE2 downregulation during viral infection, leading to cardiovascular-related complications like ARDS and AKI. In this work, the enzymatic activity attribute was of particular interest. Due to ACE2’s relatively weak binding affinity for viral spike proteins, its pan-specificity attribute was largely deferred to other modules that could more efficiently perform that function. Hence, we used naturally homodimerized ACE2_ECD_ and fused it to VHH_Spike_-optimized anti-spike nanobody modules and demonstrated broader cross-reactivity across a comprehensive panel of viral spike protein variants. These strong binding effects ensured elevated levels of virus neutralization, further demonstrating the value of adding an optimized anti-spike VHH module to the ACE2 ectodomain.

It is tempting to speculate on the molecular mechanisms underlying the observed binding cross-reactivities and neutralization potencies. In the absence of solid structural evidence describing the binding modes between the viral spike protein and the designed multifunctional fusions, several possible molecular binding architectures can be envisioned, including interactions with a single S-protein trimer or bridging between S-protein trimers. The designed multifunctional fusions are homodimers, with a total of four modules able to interact with the S protein: two VHH_Spike_ and two ACE2_ECD_ modules. While ACE2_ECD_ binds weakly to the RBD region of the S protein, the VHH_Spike_ modules tested here target either the RBD (partially overlapping with ACE2_ECD_ binding site), the NTD, or the S2 regions. The most probable binding mode for the designed homodimeric fusions is a bivalent binding in which the two VHH_Spike_ modules bind simultaneously on the same S-protein trimer. This is due to (i) the designed spacers between the VHH_Spike_ and ACE2_ECD_ modules (Table S2), (ii) the higher affinity of the VHH_Spike_ modules relative to the ACE2_ECD_ module for the S protein (26, 27), and (iii) the incompatibility of the ACE2_ECD_ homodimer to bind bivalently to the same S-protein trimer (Fig. S1). It is also worth noting that the binding modes to the S protein for each utilized VHH_Spike_ module were inferred with high reliability, either from crystallographic data (28), or from AF3 predictions matching surprisingly well the HDX-MS based epitope mapping data (Fig. S2) (26). A second, less probable but still plausible, binding mode would involve the binding of one VHH_Spike_ and one ACE2_ECD_ module of the homodimeric fusion to the same S-protein trimer. This binding configuration might occur for the VHH_Spike_ modules that bind to the RBD or NTD regions, and not for the VHH_Spike_ that binds to the S2 region (see required spacers in Table S2). In addition to intra-S-protein trimer binding modes, one can also speculate on several inter-S-protein trimers binding geometries of the homodimeric fusions, in which their four S-protein binding modules would engage adjacent spike trimers displayed on the virus surface. Obviously, the feasibility of inter-S-protein binding modes will depend strongly on the density of surface S proteins and the ability of engineered spacers to bridge between adjacent S-protein trimers. It is plausible that a certain fraction of the inter-S-protein bridging can be achieved, either on the same virus particle or between distinct viral particles. This could give rise to strong binding avidity effects and lead to potent virus neutralization and even agglutination.

Even when utilizing broadly reactive sdAbs as the VHH_Spike_ module, the spike protein of SARS-CoV-2 has the ability to evolve into numerous new variants that can escape the binding of specific VHH_Spike_ sdAbs reported in this proof-of-concept study. A glimpse of this potential decline in neutralization capacity against new variants was apparent from both the S binding (Fig. 4) and pseudovirus neutralization (Fig. 5) data for the lead compound 72opt-ACE2-R28. Indeed, up to the first Omicron variant, 72opt-ACE2-R28, with the VHH_Spike_ targeting the RBD, showed consistently stronger binding and neutralization IC_50_ values against the ancestral, Delta B.1.617.2, and Omicron B.1.1.529 strains relative to other constructs. However, the fast-evolving rate of mutations of the virus leading to more complex variants of Omicron impacted its performance. Omicron BA.4/5 and subsequent variants had a deep impact on the potency of this RBD-targeting lead construct. The severity of the decline is likely correlated with the specific recognition by a given VHH_Spike_ of the corresponding S protein and its escape mutations at the epitope targeted by the sdAb. In this regard, it is informative that the designed construct targeting the NTD region with SR01 was less affected, which is partially in agreement with the virus mutations taking place mainly on the RBD of the S protein, although the potential anti-viral efficacy of this construct against the most recent variants of concern remains to be explored. In fact, the SR01-ACE2-R28 construct even slightly improved binding (Fig. 3) and neutralization (Fig. 5) against the more recent Omicron variants relative to the ancestral strain. Noteworthy, all tested constructs showed at least some minimal level of improvement in potency against most virus variants tested relative to an ACE2_ECD_-based control lacking the VHH_Spike_ module (Fig. 4 and 5). In the few cases in which VHH_Spike_ afforded no improvement and hence failed to recognize a particular S protein, binding and neutralization were deferred solely to the ACE2_ECD_ module, which can always provide a “basal” level of natural broad specificity against all SARS coronavirus variants. Based on all these observations, several mitigation strategies against potential losses in specificity for emerging viral strains are available within the framework of the VHH_Spike_-ACE2_ECD_-VHH_Albumin_ multifunctional design. First, the combination of as few as two well-chosen constructs can significantly expand the specificity spectrum. Second, the modular scaffold allows replacing the nanobodies used in this proof-of-concept study with future VHH_Spike_ antibodies updated for broad specificity against the most current virus strains. Third, incorporating VHH_Spike_ variants against epitopes with lower rates of mutation may better sustain neutralization against future emerging strains. Lastly, the retention of the ACE2_ECD_ module from the natural entry receptor affords a basal level of binding and neutralization against the entire spectrum of past, current, and future strains.

The other addition to the ACE2_ECD_-based design described here was the fusion of an albumin-binding module, VHH_Albumin_, which demonstrated a beneficial effect on prolonging *in vivo* circulation. A particular VHH_Albumin_ was selected for this proof-of-concept study, but several other options suitable in terms of cross-species reactivities among rodents and primates and available humanized versions are also available (23). Due to different requirements for each *in vivo* experiment, we employed multiple rodent models to address model-specific limitations relevant to the biological effects under investigation. While binding of VHH_Albumin_ has been confirmed for mouse and rat serum albumins (animal models used here for PK and blood pressure experiments, respectively), albumin binding was not determined in hamster (the animal model we used for infection neutralization). However, the prolonged antiviral effect observed in SARS-CoV-2-infected hamsters for up to 5 days after single-dose administration of the VHH_Spike_-ACE2_ECD_-VHH_Albumin_ is consistent with the duration of blood pressure reduction observed in normotensive mice, as well as the persistence of ACE2 enzymatic activity in the serum from rats injected with the same single-dose of VHH_Spike_-ACE2_ECD_-VHH_Albumin_. Furthermore, binding affinities to primate serum albumins are similar to those observed in rodents (23), supporting expectations for favorable profiles in humans within similar timeframes.

There are several technical hurdles with measuring blood pressure in validated animal models of SARS-CoV-2 infection, of which the most relevant is the Syrian hamster model. Whereas in mice and rats, indirect blood pressure methods have been extensively validated and are widely accepted, the same cannot be inferred for hamsters. We attempted to use a device that indirectly calculates blood pressure by measuring pulses through the paw, but this was highly inaccurate when used with hamsters. As such, direct catheterization of arteries or telemetry probe implantation is still required to confidently measure the blood pressure in these animals. There are also significant challenges to performing surgery in hamsters, as they are more sensitive to tissue anoxia than rats, and procedures need to be performed within 2 min or necrosis of the lower extremities occurs. Furthermore, these experiments would also have to be performed inside the biosafety level-3 laboratory setting for SARS-CoV-2-infected animals, which severely adds to the complexity of this task and diminishes its feasibility. Therefore, to emulate the increase in blood pressure due to Ang-II accumulation as a result of ACE2 receptor downregulation in SARS-CoV-2-infected subjects (35), we employed an established Ang-II-induced hypertensive mouse model (36), which recapitulates persistent activation of the renin-angiotensin-aldosterone system (RAAS) and endothelial injury reported among patients with COVID-19, which are both associated with blood pressure elevation (37).

A single dose of the lead multifunctional construct in Ang-II-induced hypertensive mice had a marked effect of normalizing the systolic blood pressure. However, this effect was transient, suggesting that daily dosing may be required during the acute phase of infection in order to maintain blood pressure normalization. In healthy, normotensive animals used as controls, a single administration of the compound resulted in a 4 day duration of blood pressure reduction, but with a more modest reduction (15 mmHg) than in hypertensive animals (60 mmHg). Hence, it appears that the magnitude and duration of cardiovascular-related effects elicited by VHH_Spike_-ACE2_ECD_-VHH_Albumin_ are optimal for matching a typical acute coronaviral infection manifested by increased blood pressure. It will be interesting to understand if the profile of the present multifunctional design could also be suitable for long COVID, for which no therapy is currently available (38, 39). Although the normalization of high blood pressure in SARS-CoV-2-infected patients is the desired effect, a noteworthy related aspect is whether the modest reduction of systolic blood pressure induced by the designed constructs in the normotensive animals could lead to potential side effects due to hypotension. First, it should be mentioned that these compounds are not intended for use in healthy patients. Second, given the importance of the RAAS axis to the control of blood pressure, numerous therapies have been evaluated against this system for cardiovascular and renal protection. The recombinant ACE2_ECD_ has been tested previously in healthy patients with doses ranging from 100 mg/kg to 1,200 mg/kg through IV infusion (https://clinicaltrials.gov/study/NCT00886353) (13). In those studies, no changes in blood pressure or heart rate were observed at the end of the infusion and up to 24 h post-infusion. Under physiological conditions, blood pressure is tightly regulated, and the results from those studies potentially suggest that in healthy subjects, feedback mechanisms, such as the autonomic nervous system or renal control, counteract the effect of recombinant ACE2_ECD_, and as such, it is generally considered safe for administration. It should be noted that in our study, we injected a dose of 10 mg/kg in mice, which roughly translates to a dose of 810 mg/kg in humans (40). Since in the referenced study, even at 1,200 mg/kg, ACE2_ECD_ failed to produce any cardiovascular complications, we do not expect any major cardiovascular complication arising from our therapy based on VHH_Spike_-ACE2_ECD_-VHH_Albumin_.

Scaled-up bio-manufacturability of the proposed VHH_Spike_-ACE2_ECD_-VHH_Albumin_ general scaffold would be an important consideration for eventual clinical development. In the current study, we achieved excellent protein yields (up to 350 mg/L) using a transient CHO cell expression method, so we expect that CHO clones could be developed with sufficient yields for cost-effective drug manufacturing. The affinity tags used for purification would be inappropriate for clinical development, but the good productivity should facilitate development of an alternative downstream process. We note that the VHH modules may allow the use of industrial-grade affinity purification such as Protein A chromatography. However, in our experience with ACE2_ECD_-containing compounds (ACE2-Fc fusions and the present designs alike; data not shown), acidic pH elution from Protein A columns led to extensive protein aggregation and would not be usable as-is as a purification step for manufacturing.

Another consideration related to manufacturability is the presence of the faster-eluting “shoulder” (Peak 1) observed by UPLC-SEC for the VHH_Spike_-ACE2_ECD_-VHH_Albumin_ fusion proteins (Fig. 3). We do not believe this peak represents aggregates or multimers, given the MALS molecular weight estimate being very similar to the main peak. Also, experiments with PNGase F treatment indicate that it is not caused by differential glycosylation (Fig. S3). Instead, we suspect that conformational heterogeneity, specifically of the VHH modules, likely explains this secondary peak. Supporting this explanation, Peak 1 is detectable at low levels for the control ACE2_ECD_ construct without any fused VHH (peak area 9.8% relative to main peak), increasing to 13.4% for the fusion to a single VHH and an average of 21.6% for the dual-VHH fusions. Since the VHHs are attached to the core ACE2_ECD_ module via flexible linkers, this limited heterogeneity may be unavoidable.

Our view is that the therapeutic modality proposed here adds to, rather than competes with, the arsenal of other modalities that have been proposed based on the ectodomain of the host receptor for viral entry. A plethora of ACE2-centric construct designs can be found in the published literature (41). These include variations at the level of the ACE2_ECD_ module itself, in which the catalytic domain has been utilized alone or together with the downstream homodimerizing Neck domain, and other ACE2_ECD_ variations such as the wild-type, spike-binding affinity-improved, or enzymatically impaired versions. To increase half-life in circulation, these ACE2_ECD_ variations were fused mainly to the N-termini of Fc fragments (either fully competent, attenuated, or completely impaired for FcγR binding) (11, 12, 16–18, 42), or to the C-termini of Fc fragments in the context of full-length anti-spike antibodies (43). In other designs, ACE2_ECD_ has been fused to serum albumin or small albumin-binding domains (14, 20, 21). The alternative albumin route aimed at eliminating potentially unwanted side effects like antibody-dependent enhancement and inflammation associated with Fc-fusions via engaging the FcγRs and C1q complement complex (19, 44, 45). It became evident that each of these many ACE2-centric design platforms offered pros and cons in terms of molecular design, anti-viral efficacy, mitigation of cardiovascular effects, triggering immune receptor function and immunogenicity, toxicity, and/or scale-up manufacturability. Our novel ACE2-based scaffold was designed to satisfactorily address major drawbacks in most or all of these aspects, and data presented here at the proof-of-concept level demonstrated an overall encouraging performance, with additional refinements to be explored in a subsequent developmental stage.

In conclusion, here we described a VHH_Spike_-ACE2_ECD_-VHH_Albumin_ general scaffold, a multi-functional design engineered to achieve a wide and strong pan-coronaviral neutralization and mitigation of cardiovascular-related complications ensuing from SARS-CoV-2 infection. We showed that this construct maintains therapeutically relevant effects over an intermediate timeframe appropriate for treatment of typical acute SARS-CoV-2 infections. A few examples have been selected that form a reasonable starting point for neutralization of emerging virus variants, with the possibility of updating component modules in a plug-and-play mode. Large-scale manufacturability has to be further examined for a serious consideration of this multifunctional design toward clinical development.

## MATERIALS AND METHODS

### Structure-based design

Molecular docking of VHHs to S-protein domains was carried out using the AF3 method (29) and server (https://alphafoldserver.com/). Flexible Gly-Ser linkers were built in Sybyl 8.1.1 (Tripos, Inc., St. Louis, MO, USA). Visualization was done in PyMol (Schrödinger, Inc., New York, NY, USA).

### Protein production

The DNA sequences encoding ACE2_ECD_ alone and VHH_Spike_-ACE2_ECD_-VHH_Albumin_, VHH_Spike_-ACE2_ECD_, and ACE2_ECD_-VHH_Albumin_ fusions, codon-optimized for expression in CHO cells, were synthesized and cloned into pTT5 plasmid (46) by Twist Bioscience (South San Francisco, CA, USA). Following the IL-10 signal peptide MHSSALLCCLVLLTGVRA, the sequence EGWSHPQFEKGGGSGGGSWSHPQFEKGHHHHHHGDYKDDDDK, which contains a Twin-Strep-tag (47), a 6×His tag, and a FLAG tag, was introduced at the N-termini of the mature protein constructs for purification and detection purposes. Productions were carried out by transient transfection of CHO^55E1^ cells as described previously (48), at culture volumes of 100 mL or 500 mL. Cultures were harvested at day 7 post-transfection. Cell density and viability were monitored during the production periods by direct counting of cell samples with a Cedex automated cell counting system (Roche Innovatis, Bielefeld, Germany) using the trypan blue dye exclusion method. Post-transfection cell densities were between 1.4 and 2.8 × 10^7^ cells/mL, with cell viabilities greater than 95%.

### Purification and characterization of ACE2 constructs

Cell cultures were harvested by centrifugation at 3,300 × *g* for 30 min, and supernatants were then filtered using 0.2 µm Stericup vacuum filtration units (MilliporeSigma, Burlington, MA, USA) with a glass fiber pre-filter. Initial purifications from cell-culture supernatants were performed by IMAC, using 5 or 24 mL Ni Sepharose EXCEL columns (Cytiva, Uppsala, Sweden, Cat# 17371201) depending on the production scale. Columns were equilibrated in HyClone Dulbecco’s phosphate-buffered saline (DPBS), pH 7.4 (Cytiva, Marlborough, MA, USA). Supernatants were loaded by gravity at 2 mL/min–3 mL/min. Columns were washed with 10 mM imidazole (in DPBS containing 300 mM NaCl), and elution was performed with 300 mM imidazole (in DPBS containing 300 mM NaCl). Following SDS-PAGE analysis (NuPAGE 4–12% Bis-Tris gel, Bio-Rad XT MES running buffer, 200 V, 50 min; Invitrogen, Carlsbad, CA, USA), fractions containing the fusion protein were pooled and loaded on 15 mL StrepTactin XT Superflow high-capacity columns (IBA Lifesciences, Göttingen, Germany) for a second round of affinity purification. Columns were equilibrated with 100 mM Tris-HCl, 150 mM NaCl, pH 8.0 buffer, and washed with the same buffer. Elution was done with 100 mM Tris-HCl, 50 mM D-biotin, 1 mM EDTA, pH 8.0 buffer, and analyzed by SDS-PAGE. Protein-containing fractions were pooled and buffered-exchanged against DPBS using NAP-25 desalting columns (Cytiva, Uppsala, Sweden, Cat# 17085202) and sterilized by filtration through 0.2 μm filters. Ultra-high-performance liquid chromatography-size-exclusion chromatography (UPLC-SEC) performed on a BEH-200 column (Waters, Milford, MA, USA) was used to assess the purity of all eluates, with molecular weights of eluted protein peaks determined by MALS as described previously (49). Samples were concentrated by ultrafiltration using the Amicon Ultra-15 centrifugal concentrator (Fisher Scientific, Waltham, MA, USA) with a membrane molecular weight cut-off of 30 or 50 kDa at room temperature following the manufacturer’s instructions. During the process, the protein concentration was monitored by measurement of absorbance at 280 nm and the calculated specific extinction coefficient of each variant. Formulated samples for *in vivo* studies had endotoxin levels below 0.12 EU/mg, as measured using the Endosafe system and FDA-licensed cartridges (Charles River Laboratories, Laval, QC, Canada).

### Deglycosylation using peptide-N-glycosidase F (PNGase F)

The PNGase F enzyme was produced in-house. Briefly, a DNA sequence encoding PNGase F of *Elizabethkingia meningoseptica* (protein sequence accession no. AAA24932) was cloned into plasmid pCWori+(-lacZ). The sequence (PNG-03) encodes amino acids 41–354 (i.e., without the 40-amino-acid leader peptide) with the addition of a C-terminal 6-His tag. The enzyme was produced in *Escherichia coli* Origami BTM (Novagen, Madison, WI, USA) and purified using Ni-NTA agarose resin (Qiagen, Toronto, ON, Canada).

For removal of N-glycans, purified ACE2 constructs (1 mg/mL final concentration) were mixed with PNGase F (16.6 µg/mL final concentration) in PBS and incubated overnight at 37°C. Deglycosylated proteins, along with untreated controls, were analyzed by SDS-PAGE and UPLC-SEC as described above.

### Coronaviral cross-reactivity assessment by ELISA

Recombinant S proteins from Sarbecoronaviruses and other zoonotic coronaviruses were produced and purified as previously described (50, 51). Table S4 includes details of how each of them was produced and purified. The SDS-PAGE analyses for spike proteins that were used are assembled into Fig. S4, and for several of the spike proteins, SDS-PAGE results have been shown in previous publications as listed and referenced in Table S4. Briefly, to perform ELISA, spike proteins, shown to be fully intact by SDS-PAGE, were passively adsorbed onto NUNC Immulon 4 HBX microtiter plates (Thermo Fisher, Ottawa, ON, Canada, Cat no. 3855) at 50 ng/well in 100 µL of PBS, overnight at 4°C. The following day, plates were blocked with PBSC (1% [wt/vol] casein [Sigma, Oakville, ON, Canada, Cat no. E3414]) for 1 h at room temperature (RT). The multifunctional ACE2/VHH fusions were diluted to 250, 50, or 12.5 ng/mL in PBSC containing 0.2% (wt/vol) casein and 0.1% (vol/vol) Tween-20 for 1 h with agitation at 300 rpm. Plates were then washed five times with PBST (PBS supplemented with 0.05% [vol/vol] Tween-20) and incubated with 100 µL/well of 800 ng/mL of horseradish peroxidase-conjugated anti-VHH domain antibody (AffiniPure Goat Anti-Alpaca IgG, Jackson ImmunoResearch, West Grove, PA, USA Cat no. 128-035-232) for an additional hour. Finally, following five washes as described above, 100 µL of peroxidase substrate solution (BD OptEIA TMB Substrate Reagent, BD Biosciences, Franklin Lakes, NJ, USA, Cat no. 555214) was added, and the plates were incubated for 15 min at RT. The reaction was stopped by adding 50 µL/well of 1 N H_2_SO_4_, and absorbance was subsequently measured at 450 nm using a Multiskan FC photometer (Thermo Fisher, Ottawa, ON, Canada).

### *In vitro* pseudovirus neutralization

Pseudotyped SARS-CoV-2 spike lentiviral particles were produced using either the pHDM-SARS-CoV-2-Wuhan-Hu-1 (ancestral strain) expressing the SARS-CoV-2 Wuhan-Hu-1 S protein (GenBank no. NC_045512), pcDNA3.3-SARS-CoV-2-B.1.617.2 expressing the SARS-CoV-2 B.1.617.2 (Delta variant) S protein (a gift from David Nemazee, Addgene plasmid no. 172320) (52), pTwist-SARS-CoV-2-Δ18-B.1.1.529 expressing SARS-CoV-2 B.1.1.529 (Omicron variant) S protein (a gift from Alejandro Balazs, Addgene plasmid no. 179907) (53), or pCAGGS-SARS-CoV-2-BA.4/5 expressing SARS-CoV-2 BA.4/5 (Omicron variant) S protein (a gift from Marceline Côté, Addgene plasmid no. 186031) (54), under a CMV promoter, and packaged into lentiviral vectors obtained through BEI Resources, NIAID, NIH: SARS-Related Coronavirus 2, Wuhan-Hu-1 Spike-Pseudotyped Lentiviral Kit (NR-52948). Productions were performed according to the protocols and reagents described previously (33), with the following modifications: (i) HEK293SF-3F6 cells (55) were used for large-scale production of lentiviral particles in 300 mL; (ii) post-transfection HEK293SF-3F6 cells were incubated at 33°C for improved yield; and (iii) 72 h post-transfection, lentiviral particles were harvested and concentrated by sucrose cushion centrifugation. Briefly, the supernatant was placed on a 20% sucrose cushion and spun for 3 h at 37,000 × *g* at 4°C. The pellet containing the concentrated pseudotyped virus-like particles was then resuspended in DMEM containing 10% fetal bovine serum and aliquoted. Titration was performed using HEK293T cells overexpressing human ACE2 and TMPRSS2, obtained from BEI Resources repository of ATCC and the NIH (NR-55293). Pseudovirus neutralization assay was adapted for 384-well plates. Briefly, threefold serial dilutions of samples containing test fusion proteins or control antibodies (REGN10933 [34] and human isotype IgG) were incubated with diluted virus at a 1:1 ratio for 1 h at 37°C before addition to HEK293T-ACE2/TMPRSS2 cells. Infectivity was then measured by luminescence. Bright-Glo luciferase reagent (Promega, Madison, WI, USA, Cat no. E2620) was added to wells for 2 min before reading with a PerkinElmer Envision instrument. Half-maximal inhibitory concentrations (IC_50_s) were calculated based on a variable-slope nonlinear regression model between the normalized response (% infection) versus the logarithm of inhibitor concentration, using Prism 8 (GraphPad Software Inc., Boston, MA, USA).

### *In vivo* pharmacokinetics

Male Wistar rats (5–6 weeks old) were obtained from Charles River Laboratories (Saint-Constant, QC, Canada). Two groups of three animals each were injected intravenously via the tail vein with a single dose of 10 mg/kg of either 72opt-ACE2-R28 or 72opt-ACE2 fusion proteins diluted in PBS. Two naïve animals were used as reference for the collection of baseline (untreated) blood. Blood samples were collected from the tail vein at time intervals of 0.25, 1, 3, 6, 24, 48, 72, and 96 h post-administration. To obtain serum, the blood samples were allowed to clot at RT for 15–30 min and then centrifuged at 1,500 × *g* for 10 min at RT. The serum was carefully separated and immediately transferred into pre-labelled tubes (~40 µL per tube) for ACE2 activity analysis. Serum samples were snap-frozen on dry ice and stored at −80°C until analysis.

The ACE2 enzymatic activity was measured as previously described (56). Experiments were performed in black 96-well plates (Corning, Tewksbury, MA, USA, Cat no. 3915). Briefly, various volumes of rat sera were incubated with 75 µL ACE2 substrate-assay buffer (15 µM ACE2 substrate Mca-Ala-Pro-Lys(Dnp)-OH, 1 mM N-ethylmaleimide, 1 mM phenylmethylsulfonyl fluoride, 50 mM 2-(N-morpholino)-ethanesulfonic acid monohydrate, 300 mM NaCl, 10 µM ZnCl_2_), and the kinetic profile was obtained by readings every 5 min at RT. The fluorescence (excitation wavelength of 320 nm, emission wavelength of 405 nm, and a filter bandwidth of 10 nm) was measured with a BioTek Cytation5 plate reader (Agilent, Santa Clara, CA, USA). The calculated ACE2 activities (RFU/min/µL) in serum samples were corrected for the equivalent volumes of background serum-only controls.

For each individual, a linear/log trapezoidal method was employed to estimate the AUC of the serum ACE2 enzymatic activity versus time, from time zero to the last observed quantifiable time point (*AUC_0-96_*) using Phoenix WinNonlin 8.5 (Pharsight Corporation, Mountain View, CA, USA).

### *In vivo* infection neutralization efficiency

Male Syrian golden hamsters (81 g–90 g) were obtained from Charles River Laboratories (Saint-Constant, QC, Canada). Animal health was monitored daily, and clinical scores were determined. Animals were euthanized by exposure to CO_2_. Animals were anesthetized under ketamine/xylazine then challenged intranasally with 10^4^ PFUs of SARS-CoV-2 isolate Canada/ON/VIDO-01/2020. Four hours post-challenge, animals were administered intravenously via the retro-orbital vein with 150 µL of 72opt-ACE2-R28 fusion at a single dose of 3 mg/kg or 10 mg/kg, in cohorts of seven animals for each dose. A control cohort of seven animals was administered 150 µL of PBS vehicle. Daily body weights were recorded. Animals were euthanized at 5 days post-challenge, and lung tissues were collected.

### Immunohistochemistry

Lung tissues collected from the infected hamsters were fixed in 10% neutral buffered formalin for 1 week at RT, processed, and embedded in paraffin wax using standard protocols. The paraffin block was cut in 5 µm-thick sections and placed on Superfrost Plus slides (Fisher Scientific, Waltham, MA, USA). Sections were dried overnight and then subjected to IHC using a modified protocol F on the Bond-Max fully automated system (Leica Biosystems, Wetzlar, Germany). All reagents from the Bond Polymer Refine Detection Kit (DC9800) were used for IHC. A mouse monoclonal antibody against the SARS-CoV-2 N protein (1:5,000, R&D Systems, Minneapolis, MN, USA, Cat no. MAB10474) was used for the detection of SARS-CoV-2. Following deparaffinization and rehydration, sections were pre-treated with the Epitope Retrieval Solution 1 (ER1, citrate buffer, pH 5.0) at 98°C for 20 min to expose the epitopes. After washes, non-specific endogenous peroxidases were quenched using peroxidase block for 5 min; sections were washed again and then incubated for 15 min at RT with anti-SARS-CoV-2 N antibody. Sections were incubated with polymer refine for 8 min at room temperature and developed with 3,3′-diaminobenzidine for 10 min. Sections were washed again and counterstained for 6 min with hematoxylin, dehydrated, cleared, and mounted. Negative controls included omission of primary antibody and incubation with secondary antibody alone, as well as lung tissue from naïve animals.

Following IHC, whole lung sections were scanned at 20× magnification using a ZEISS Axioscan.Z1 digital slide scanner capable of brightfield imaging (Zeiss, Oberkochen, Germany). QuPath 0.3.2, an open-source software for bioimage analysis (https://qupath.github.io) (57), was used to detect and count immune-positive cells in whole slide sections as described previously (58). The number of positive cells and total area were used to calculate the average number of positive cells per square millimeter of lung tissue.

### Cardiovascular effects

Male CD-1 mice (19 g–21 g, 4 weeks old) were obtained from Charles River Laboratories (Saint-Constant, QC, Canada). Animals were split into two cohorts, one of normotensive mice and the other of hypertensive mice. Each cohort consisted of two groups of seven animals each, one test group administered the fusion protein and one control group administered the PBS vehicle.

In the normotensive cohort, following 3 weeks of acclimatization, baseline levels of SBP, body weight, and spot urine profile were established 1 day before treatment. On the day of treatment, the 72opt-ACE2-R28 fusion protein was injected intravenously via the tail vein at a single dose of 10 mg/kg. SBP was measured at the tail using a high-throughput blood pressure apparatus (Kent Scientific, Torrington, CT, USA) at predetermined time intervals of 4, 24, 48, 72, 96, 120, 144, 168, and 192 h post-injection. Body weights were monitored daily. Urine samples were collected 7 days post-injection. Total protein concentration was measured by the Bradford protein assay (Bio-Rad Laboratories, Hercules, CA, USA).

In the hypertensive cohort, following 2 weeks of acclimatization, an osmotic mini-pump was implanted subcutaneously (Alzet Model 2004, Alzet, Cupertino, CA, USA). The mini-pump was used to inject Ang-II (MilliporeSigma, Oakville, ON, Canada) at a rate of 1.44 mg/kg/day (the equivalent of 1,000 ng/kg/min) for 2 weeks. During this period of establishment of the hypertensive regime, SBP measurements were taken every 3 days. On the 14th day after the beginning of Ang-II infusion, the 72opt-ACE2-R28 fusion compound was injected intravenously via the tail vein at a single dose of 10 mg/kg. SBP was measured at the tail using the high-throughput blood pressure apparatus (Kent Scientific, Torrington, CT, USA) at predetermined time intervals of 4, 24, 48, 72, 96, 120, and 144 h post-injection.

## SUPPLEMENTAL MATERIAL

Supplemental figures and tables aac.00024-26-s0001.pdf Supporting information for molecular design, production and purification, binding experiments, animal study design, amino-acid sequences, and deglycosylation experiments, for the various ACE2/VHH fusions described in this study.

ASM does not own the copyrights to Supplemental Material that may be linked to, or accessed through, an article. The authors have granted ASM a non-exclusive, world-wide license to publish the Supplemental Material files. Please contact the corresponding author directly for reuse.
