# Supplementary material for: Multifunctional ACE2-nanobody fusion design for pan-specific neutralization and cardiovascular protection in SARS coronavirus infection
Source: Antimicrob Agents Chemother. 2026 Mar 23;70(5):e00024-26. doi: 10.1128/aac.00024-26 (PMC13148018; doi:10.1128/aac.00024-26)
Supplement: Supplemental figures and tables — Supporting information for molecular design, production and purification, binding experiments, animal study design, amino-acid sequences, and deglycosylation experiments, for the various ACE2/VHH fusions described in this study. [file aac.00024-26-s0001.pdf]

Supplemental Information

for

Multifunctional ACE2-Nanobody Fusion Design for Pan-Specific Neutralization and Cardiovascular Protection in SARS Coronavirus Infection

Traian Sulea,[1,2]* Matthew Stuible,[1] Maria Moreno,[3] Alex Pelletier,[1] Martin A. Rossotti,[3] Yuneivy Cepero-Donates,[1#] Jacqueline Slinn,[3] Patrick Salois,[1#] Anh Tran,[3,4] Melissa Hewitt,[3] Alvaro Yogi,[3] Nazanin Rohani,[1#] Brian Cass,[1] Anne E.G. Lenferink,[1] Jamshid Tanha,[3,4,5] Binbing Ling,[3] Etienne Lessard,[1] Laurence Delafosse,[1] Jagdeep K. Sandhu,[3] Danica Stanimirovic[3†]

[1]Human Health Therapeutics Research Centre, National Research Council Canada, Montreal, Quebec, Canada
[2]Institute of Parasitology, McGill University, Sainte-Anne-de-Bellevue, Quebec, Canada
[3]Human Health Therapeutics Research Centre, National Research Council Canada, Ottawa, Ontario, Canada
[4]Department of Biochemistry, Microbiology and Immunology, University of Ottawa, Ottawa, Ontario, Canada
[5]Center for Infection, Immunity and Inflammation, University of Ottawa, Ottawa, Ontario, Canada

Running Head: ACE2-Nanobody Fusions as Anti-Sarbecoviral Agents

*Address correspondence to Traian Sulea, traian.sulea@nrc-cnrc.gc.ca.
[#]Present address: Medical Devices Research Centre, National Research Council Canada, Montreal, Quebec, Canada
[†]Deceased

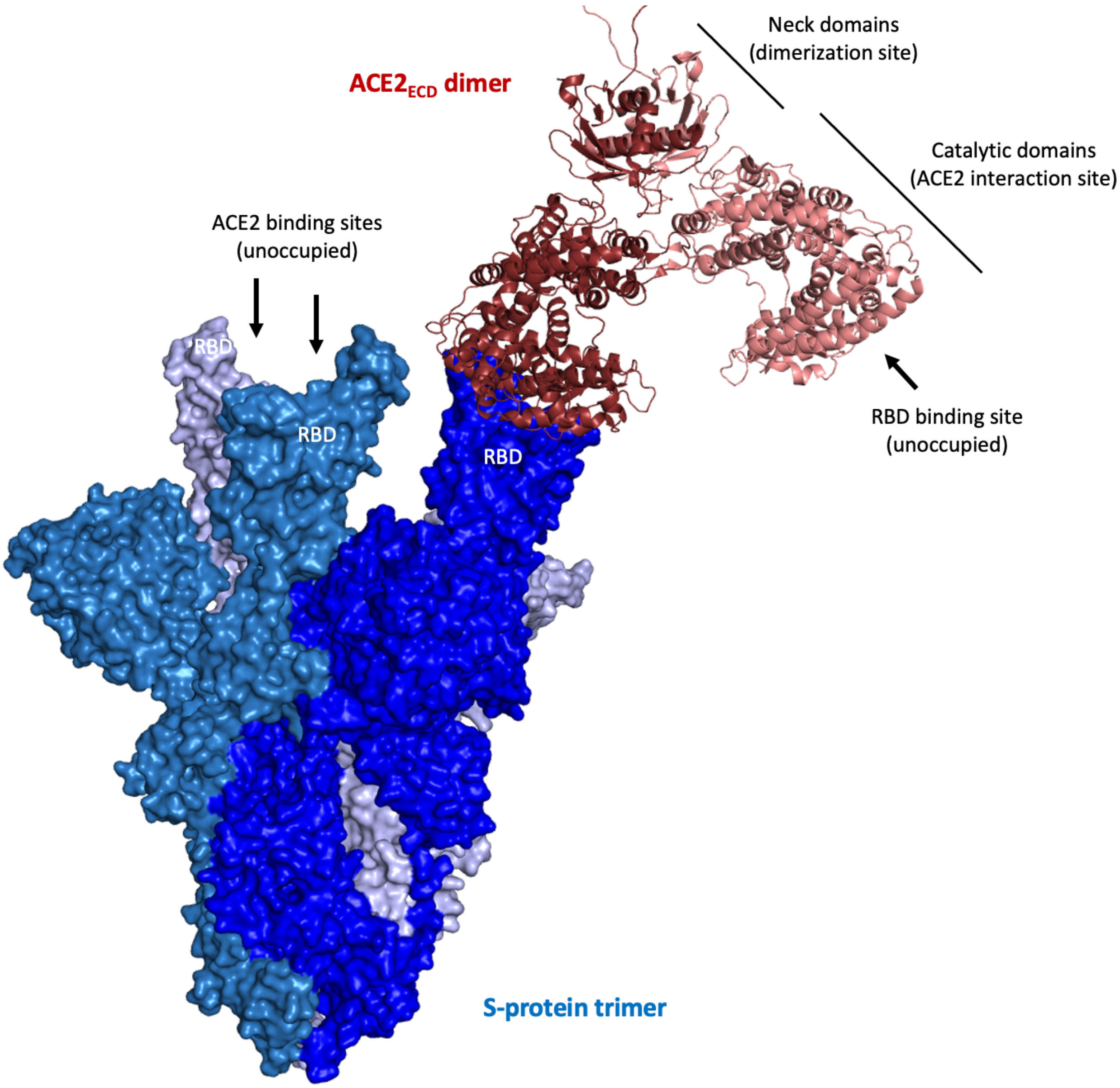

Figure S1: Geometry of $ACE2_{ECD}$ homodimer is not compatible with bivalent binding to one S-protein homotrimer. The $ACE2_{ECD}$ homodimer is shown as ribbon with monomers shown in hues of red, and S-protein homotrimer is shown as surface with monomers shown in hues of blue. One available RBD interaction site of the $ACE2_{ECD}$ homodimer is not suitably positioned to interact with either of the two available ACE2 interaction sites of the S-protein trimer given the monovalent interaction shown between ACE2 and S-protein. The image was generated starting from the ACE2 homodimer bound to the S-protein RBD (PDB code 6M17) and the structure of the S-protein trimer (PDB code 7A98).

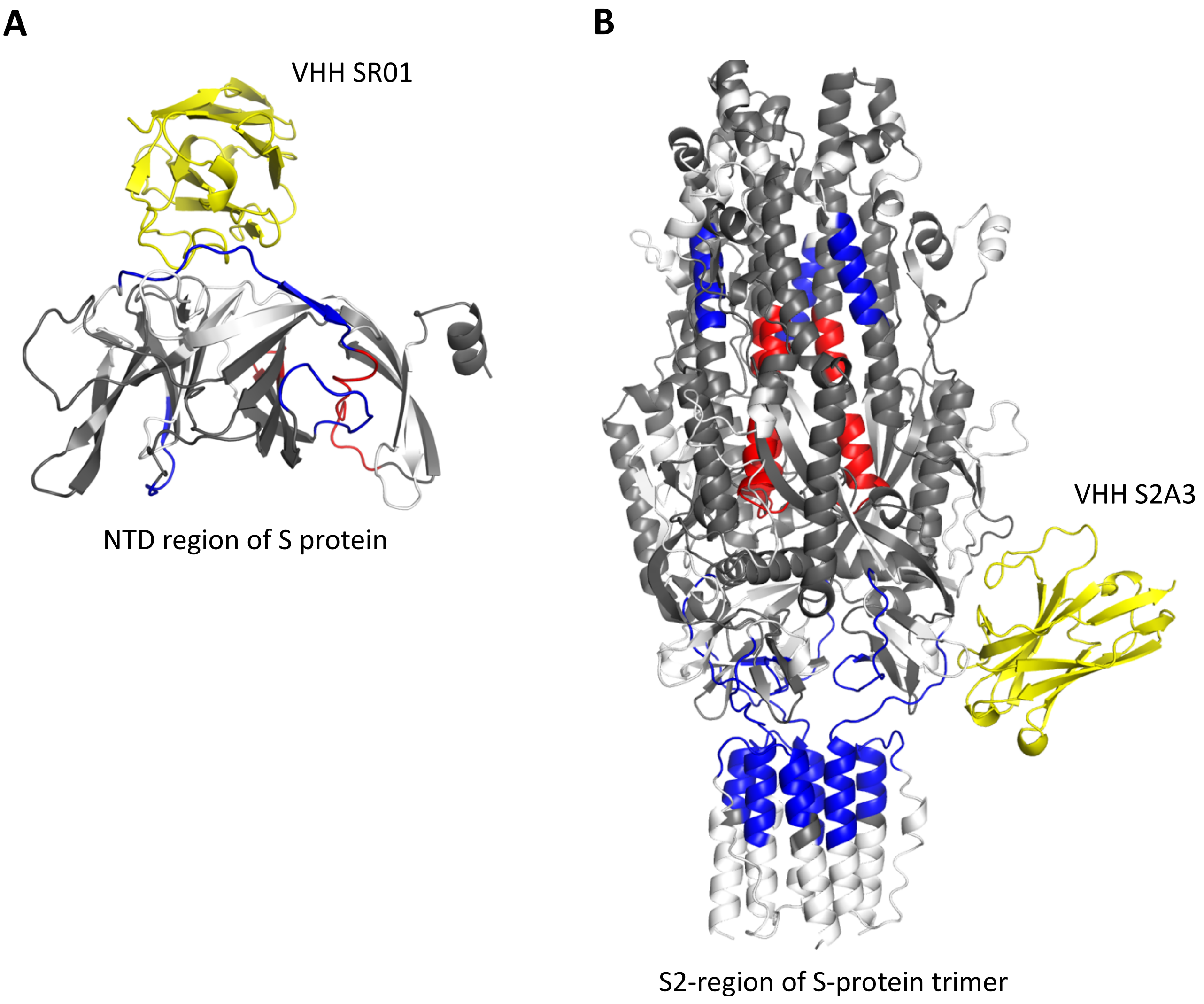

Figure S2: Mapping of experimental HDX-MS data on AlphaFold3-docking-based binding modes of VHH_{Spike} to the S protein. HDX-MS data are taken from Rossotti et al. *Commun. Biol.* (2022) 5:933. Regions on the S protein where significant changes in deuteration (> 3 SD cut-off, *p*-value 0.02) were observed upon VHH_{Spike} binding are colored blue (stabilization) and red (destabilization), while regions with no significant changes in deuteration are shown in gray and missing coverage in white. (A) VHH SR01 binding to NTD region. (B) VHH S2A3 binding to S2 region.

A

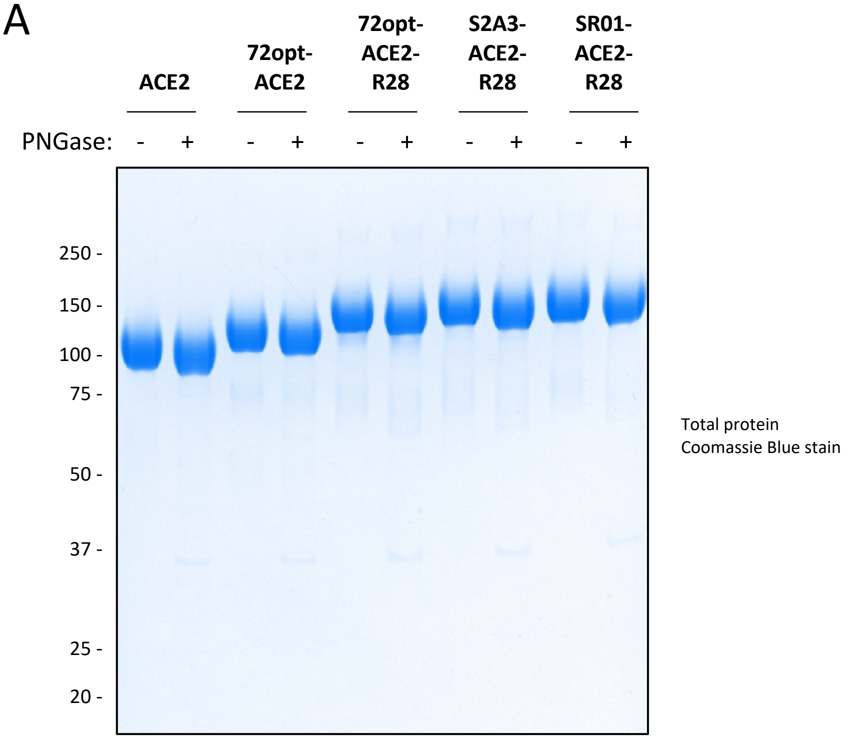

B

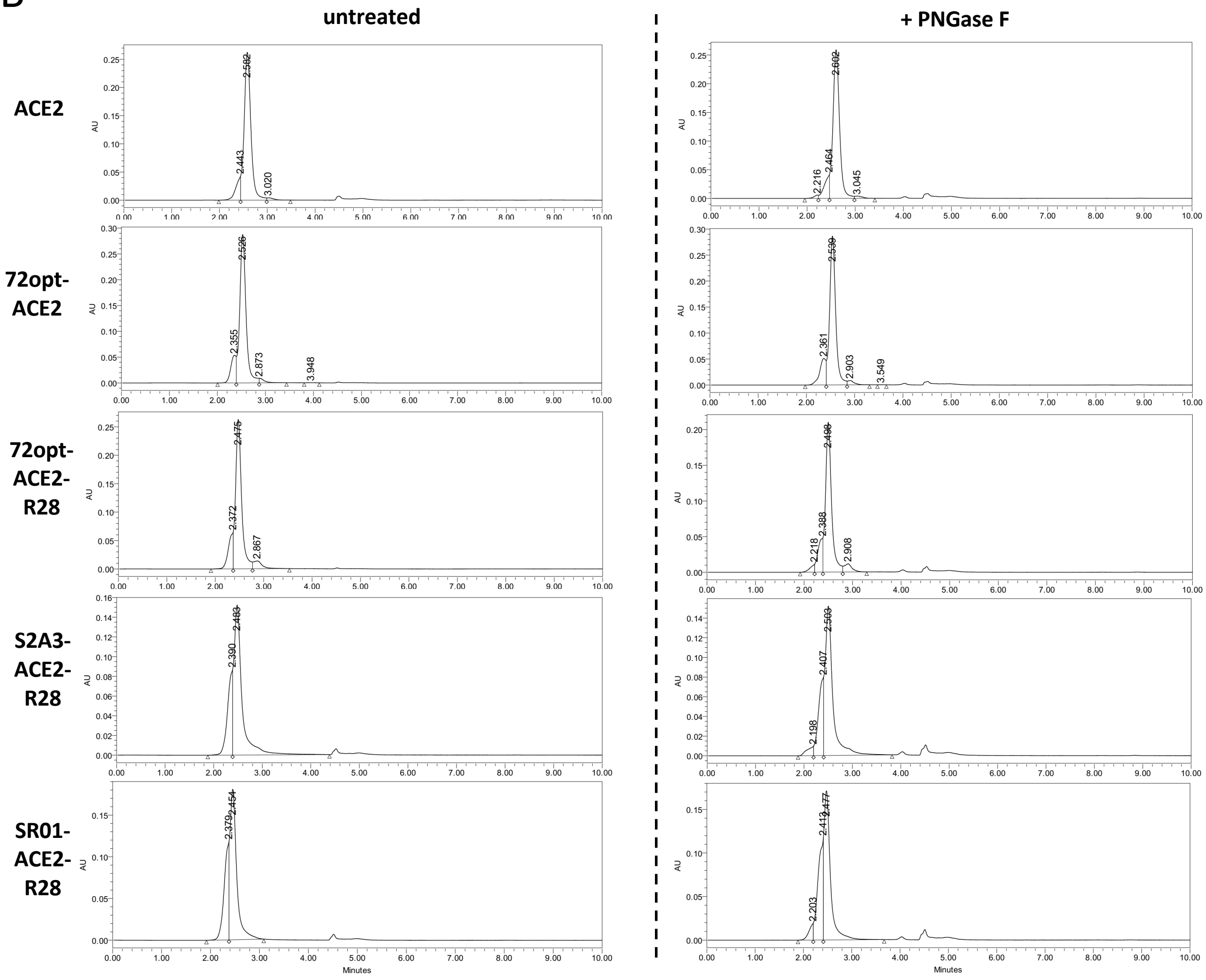

Figure S3: Effects of PNGase F-mediated removal of N-glycans on SDS-PAGE and UPLC-SEC profiles of VHH/ACE2 fusion proteins. Purified fusion proteins, untreated or treated with PNGase F, were analyzed by SDS-PAGE (A) and UPLC-SEC (B). In panel A, the numbers on the left represent molecular weight markers (kDa).

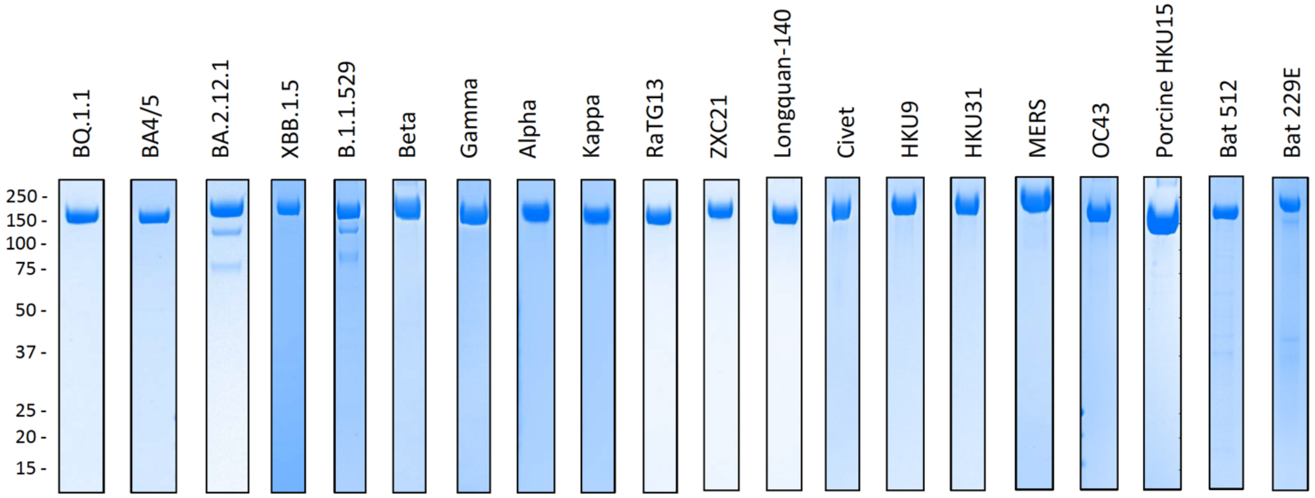

Figure S4: SDS-PAGE analysis of purified spike proteins used in ELISA (Figure 4). For spike proteins not described in previous publications (Table S4), 2-3 μg of purified protein was analyzed by SDS-PAGE followed by total protein (Coomassie Blue) staining. The numbers on the left represent molecular weight markers (kDa).

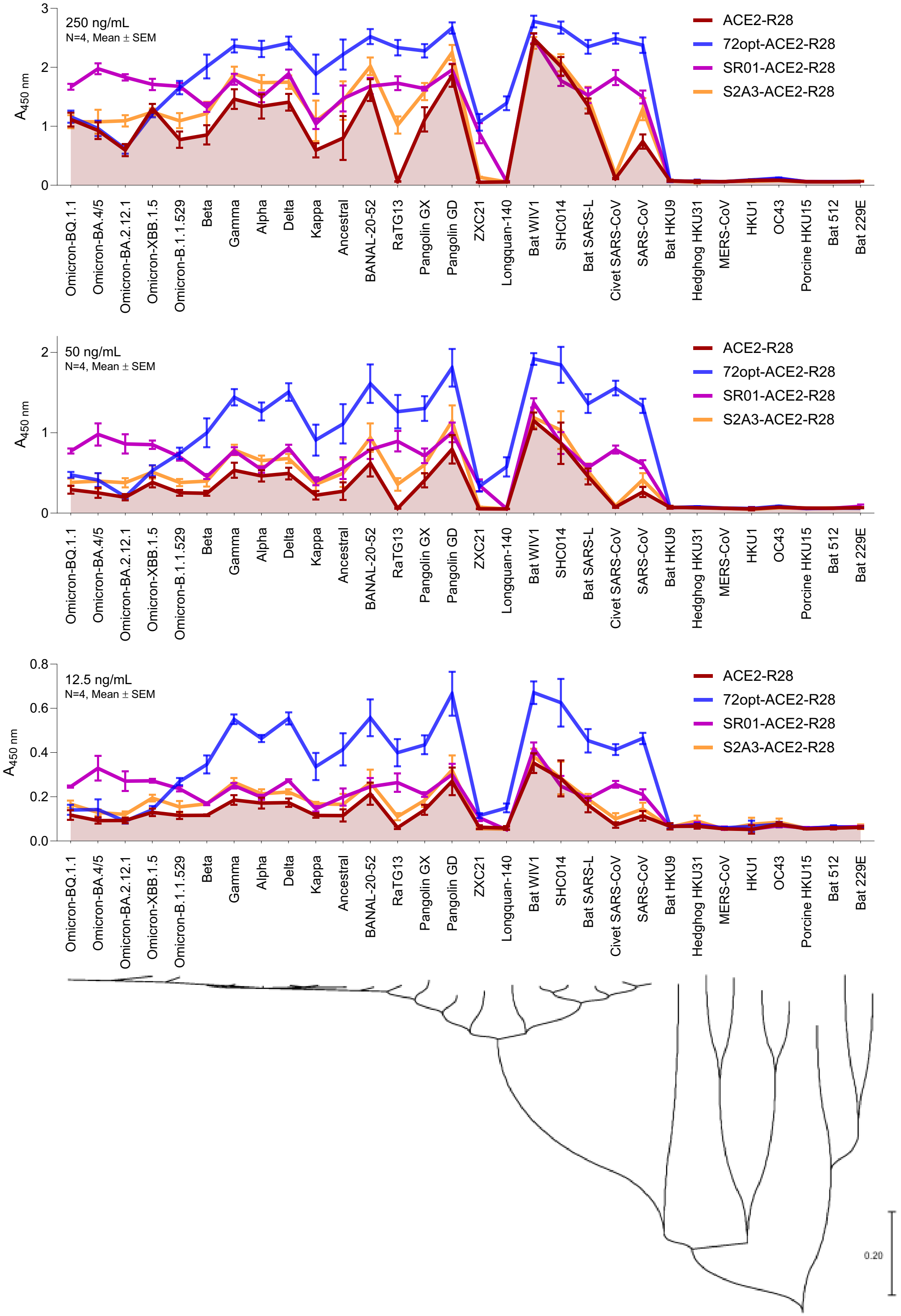

Figure S5: Cross-reactivity of VHH_{Spike}-$ACE2_{ECD}$-$VHH_{Albumin}$ fusion constructs against coronaviral S proteins determined by ELISA. Data were obtained at three different concentrations of VHH_{Spike}-$ACE2_{ECD}$-$VHH_{Albumin}$ fusion constructs: 250 ng/mL, 50 ng/mL (also featured in Figure 4), and 12.5 ng/mL. ACE2-R28 was included as reference. See Figure 4 in the main paper for additional details.

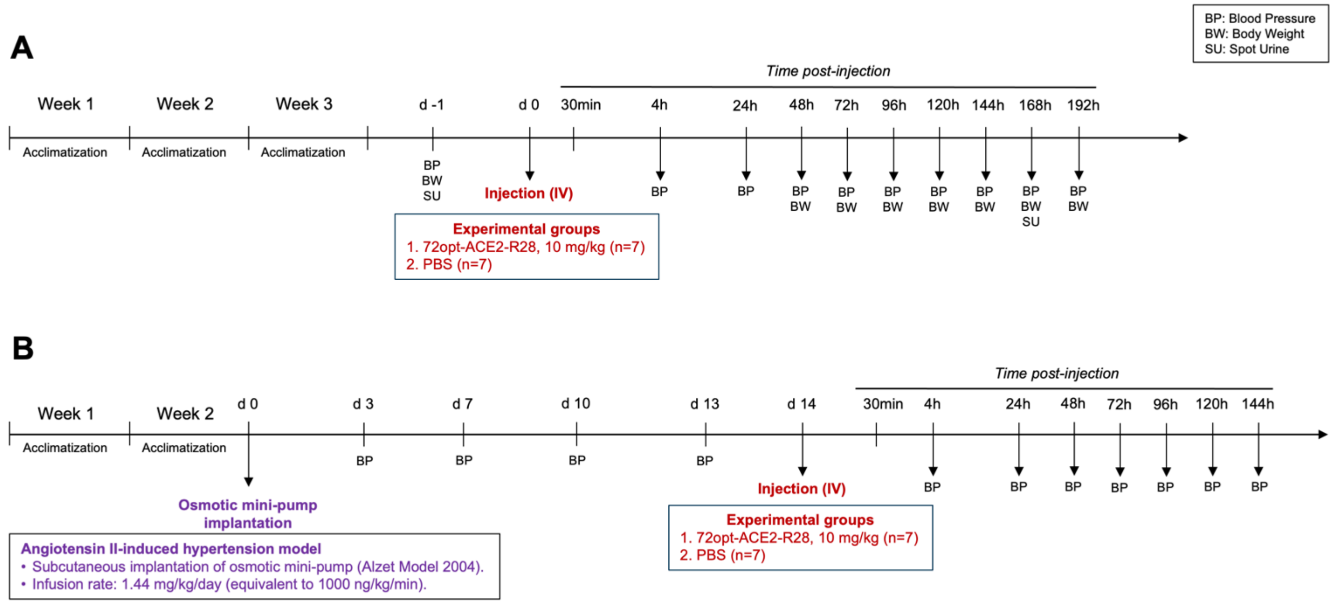

Figure S6: *In vivo* models for testing the lead compound 72opt-ACE2-R28 for cardiovascular effects and preliminary toxicity. (A) Normotensive mouse model. (B) Hypertensive mouse model. Male CD-1 mice (19-21 g, 4-weeks old) were used for the *in vivo* studies.

Table S1: Amino-acid sequences of designed and tested VHH_{Spike}-$ACE2_{ECD}$-$VHH_{Albumin}$ fusion constructs. Mature proteins lack the signal peptides.

Name	Protein sequence
72opt-ACE2-R28	MHSSALLCCLVLLTGVRAEGWSHPQFEKGGGSGGGSWSHPQFEKGHHHHHHGDYKDDDDKQVQLQESGGG<br>LVQAGGSLRLSCAASGRTFSEYAMGWFRQAPGKEREFVATISWSGGMTYYTDSVKGRFTISRDNAKNTVY<br>LQMNSLKPDDTAVYYCAAAGWGVVVSEWDYDYDYWGQGTQVTVSSGGGGSGGGGSGGGGSGGGGSGGGGQ<br>STIEEQAKTFLDKFNHEAEDLFYQSSLASWNYNTNITEENVQNMNNAGDKWSAFLKEQSTLAQMYPLQEI<br>QNLTVKLQLQALQQNGSSVLSEDKSKRLNTILNTMSTIYSTGKVCNPDNPQECLLLEPGLNEIMANSLDY<br>NERLWAWESWRSEVGKQLRPLYEEYVVLKNEMARANHYEDYGDYWRGDYEVNGVDGYDYSRGQLIEDVEH<br>TFEEIKPLYEHLHAYVRAKLMNAYPSYISPIGCLPAHLLGDMWGRFWTNLYSLTVPFGQKPNIDVTDAMV<br>DQAWDAQRIFKEAEKFFVSVGLPNMTQGFWENSMLTDPGNVQKAVCHPTAWDLGKGDFRILMCTKVTMDD<br>FLTAHHEMGHIQYDMAYAAQPFLLRNGANEGFHEAVGEIMSLSAATPKHLKSIGLLSPDFQEDNETEINF<br>LLKQALTIVGTLPFTYMLEKWRWMVFKGEIPKDQWMKKWWEMKREIVGVVEPVPHDETYCDPASLFHVSN<br>DYSFIRYYTRTLYQFQFQEALCQAAKHEGPLHKCDISNSTEAGQKLFNMLRLGKSEPWTLALENVVGAKN<br>MNVRPLLNYFEPLFTWLKDQNKNSFVGWSTDWSPYADQSIKVRISLKSALGDKAYEWNDNEMYLFRSSVA<br>YAMRQYFLKVKNQMILFGEEDVRVANLKPRISFNFFVTAPKNVSDIIPRTEVEKAIRMSRSRINDAFRLN<br>DNSLEFLGIQPTLGPPNQPPVSGGGGSGGGGSGGGGSGGGGSGGGGSQVQLVESGGGLVQAGGSLRLSCV<br>ASGRTFIAYAMGWFRQAPGKEREFVAAITNFAGGTTYYADSVKGRFTISRDNAKTTVYLQMNSLKPEDTA<br>LYYCAADRSAQTMRQVRPVLPYWGQGTQVTVSS
SR01-ACE2-R28	MHSSALLCCLVLLTGVRAEGWSHPQFEKGGGSGGGSWSHPQFEKGHHHHHHGDYKDDDDKEVQLVQSGGG<br>SVQAGGSLRLSCVASGFTFDNYAIGWFRQAPGKEREGVSCISGNGGVTIYADSVKGRFTISRDNAKNLVY<br>LQMNSLKPEDTAVYYCAATGIRSTWSVYGCSRLAGPYDYWGQGTQVTVSSGGGGSGGGGSGGGGSGGGGS<br>GGGGSGGGGQSTIEEQAKTFLDKFNHEAEDLFYQSSLASWNYNTNITEENVQNMNNAGDKWSAFLKEQST<br>LAQMYPLQEIQNLTVKLQLQALQQNGSSVLSEDKSKRLNTILNTMSTIYSTGKVCNPDNPQECLLLEPGL<br>NEIMANSLDYNERLWAWESWRSEVGKQLRPLYEEYVVLKNEMARANHYEDYGDYWRGDYEVNGVDGYDYS<br>RGQLIEDVEHTFEEIKPLYEHLHAYVRAKLMNAYPSYISPIGCLPAHLLGDMWGRFWTNLYSLTVPFGQK<br>PNIDVTDAMVDQAWDAQRIFKEAEKFFVSVGLPNMTQGFWENSMLTDPGNVQKAVCHPTAWDLGKGDFRI<br>LMCTKVTMDDFLTAHHEMGHIQYDMAYAAQPFLLRNGANEGFHEAVGEIMSLSAATPKHLKSIGLLSPDF<br>QEDNETEINFLLKQALTIVGTLPFTYMLEKWRWMVFKGEIPKDQWMKKWWEMKREIVGVVEPVPHDETYC<br>DPASLFHVSNDYSFIRYYTRTLYQFQFQEALCQAAKHEGPLHKCDISNSTEAGQKLFNMLRLGKSEPWTL<br>ALENVVGAKNMNVRPLLNYFEPLFTWLKDQNKNSFVGWSTDWSPYADQSIKVRISLKSALGDKAYEWNDN<br>EMYLFRSSVAYAMRQYFLKVKNQMILFGEEDVRVANLKPRISFNFFVTAPKNVSDIIPRTEVEKAIRMSR<br>SRINDAFRLNDNSLEFLGIQPTLGPPNQPPVSGGGGSGGGGSGGGGSGGGGSGGGGSQVQLVESGGGLVQ<br>AGGSLRLSCVASGRTFIAYAMGWFRQAPGKEREFVAAITNFAGGTTYYADSVKGRFTISRDNAKTTVYLQ<br>MNSLKPEDTALYYCAADRSAQTMRQVRPVLPYWGQGTQVTVSS
S2A3-ACE2-R28	MHSSALLCCLVLLTGVRAEGWSHPQFEKGGGSGGGSWSHPQFEKGHHHHHHGDYKDDDDKEVQLQQSGGG<br>LVQAGGSLRLSCAASGRPYSNYAMAWYRQAPGKQHELVAGKQRELVAAISSGGTTKYADSVKARFTISRD<br>NAKNTVYLQMNILRPEDTAVYYCNTGSLSYGGSVYYPSYDNWGQGTQVTVSSGGGGSGGGGSGGGGSGGG<br>GSGGGGQSTIEEQAKTFLDKFNHEAEDLFYQSSLASWNYNTNITEENVQNMNNAGDKWSAFLKEQSTLAQ<br>MYPLQEIQNLTVKLQLQALQQNGSSVLSEDKSKRLNTILNTMSTIYSTGKVCNPDNPQECLLLEPGLNEI<br>MANSLDYNERLWAWESWRSEVGKQLRPLYEEYVVLKNEMARANHYEDYGDYWRGDYEVNGVDGYDYSRGQ<br>LIEDVEHTFEEIKPLYEHLHAYVRAKLMNAYPSYISPIGCLPAHLLGDMWGRFWTNLYSLTVPFGQKPNI<br>DVTDAMVDQAWDAQRIFKEAEKFFVSVGLPNMTQGFWENSMLTDPGNVQKAVCHPTAWDLGKGDFRILMC<br>TKVTMDDFLTAHHEMGHIQYDMAYAAQPFLLRNGANEGFHEAVGEIMSLSAATPKHLKSIGLLSPDFQED<br>NETEINFLLKQALTIVGTLPFTYMLEKWRWMVFKGEIPKDQWMKKWWEMKREIVGVVEPVPHDETYCDPA<br>SLFHVSNDYSFIRYYTRTLYQFQFQEALCQAAKHEGPLHKCDISNSTEAGQKLFNMLRLGKSEPWTLALE<br>NVVGAKNMNVRPLLNYFEPLFTWLKDQNKNSFVGWSTDWSPYADQSIKVRISLKSALGDKAYEWNDNEMY<br>LFRSSVAYAMRQYFLKVKNQMILFGEEDVRVANLKPRISFNFFVTAPKNVSDIIPRTEVEKAIRMSRSRI<br>NDAFRLNDNSLEFLGIQPTLGPPNQPPVSGGGGSGGGGSGGGGSGGGGSGGGGSQVQLVESGGGLVQAGG<br>SLRLSCVASGRTFIAYAMGWFRQAPGKEREFVAAITNFAGGTTYYADSVKGRFTISRDNAKTTVYLQMNS<br>LKPEDTALYYCAADRSAQTMRQVRPVLPYWGQGTQVTVSS

Color legend: **Signal peptide**, **dual Strep peptide**, **His tag**, **FLAG tag**, **VHH_{Spike}**, **$ACE2_{ECD}$**, **$VHH_{Albumin}$**, **Linkers**

Table S2: Theoretical lengths of linkers between VHH_{Spike} and $ACE2_{ECD}$ modules required for hypothetical trivalent binding of dimeric fusion constructs to one S-protein trimer.

Dimeric fusion construct	Binding mode [a]	$VHH_{Spike}^{C\text{-}term}$ – $ACE2_{ECD}^{N\text{-}term}$ linear distances (Å) for monomer 1; monomer 2 [b]	Minimal number of residues required in flexible linker [c]	Number of linker residues used in this study
72opt-ACE2-R28	1	58; 191	23; 76	24; 24
	2	94; 123	38; 49	
SR01-ACE2-R28	1	56; 170	22; 68	29; 29
	2	57; 125	23; 50	
	3	79; 170	32; 68	
	4	57; 176	23; 70	
	5	79; 125	32; 50	
	6	56; 176	22; 70	
S2A3-ACE2-R28	1	156; 246	62; 98	24; 24
	2	175; 190	70; 76	
	3	156; 250	62; 100	
	4	160; 190	64; 76	
	5	160; 246	64; 98	
	6	175; 250	70; 100	

[a] Trivalent binding would consist of two VHH_{Spike} modules (high affinity) and one $ACE2_{ECD}$ module (low affinity) simultaneously binding to the same S-protein trimer. Six binding modes would theoretically be possible for such a trivalent binding, except for the 72opt-ACE2-R28 in which the VHH_{Spike} and $ACE2_{ECD}$ modules can compete for the same RBD domain on the S-protein trimer.

[b] For each binding mode, linear inter-atomic distances are measured between the C-terminus of the VHH_{Spike} domain and the N-terminus of the $ACE2_{ECD}$ domain. The PyMol software (Schroedinger, Inc.) was employed on AF3-docked structures of the VHH_{Spike} modules and S-protein regions (see the Materials and Methods section in the main paper, and Figure S2), which were overlayed with the experimental structure dimeric $ACE2_{ECD}$ bound to the S-protein RBD (PDB code 6M17) and the trimeric structure of the S-protein (PDB code 7A98).

[c] The linear distance divided by 2.5 Å to allow for deviations from linear path to prevent steric clashes with the rest of the protein complex.

Table S3: Peak areas (UV absorbance) for UPLC-SEC analyses of ACE2 constructs with or without PNGase F treatment. Peaks were identified in SEC chromatograms as shown in supplementary Figure 3 (Peak 1 is the high-molecular-weight peak, Peak 2 is the main peak, and LMW is the sum of any low-molecular-weight peaks).

Construct name	PNGase F treatment	Peak 1 (% area)	Peak 2 (% area)	LMW (% area)
ACE2	-	9.8	88.9	1.3
ACE2	+	11.7	86.9	3.0
72opt-ACE2	-	13.2	84.7	2.1
72opt-ACE2	+	15.1	82.2	2.7
72opt-ACE2-R28	-	15.0	78.9	6.1
72opt-ACE2-R28	+	17.1	77.4	5.5
S2A3-ACE2-R28	-	25.5	74.5	nd
S2A3-ACE2-R28	+	28.2	71.8	nd
SR01-ACE2-R28	-	31.3	68.7	nd
SR01-ACE2-R28	+	38.6	61.4	nd

nd: not detected

Table S4: Details for recombinant spike proteins used for ELISA binding assays (Figure 4). All constructs consist of the spike ectodomain fused to human resistin at the C-terminus (to mediate trimerization). Production and purification methods have been described previously (references listed in table). SDS-PAGE analyses of spike proteins not previously published are shown in Figure S4.

Spike protein name	Production method and reference	Purification method and reference	SDS-PAGE reference
Omicron BQ.1.1	CHO^{55E1} transient [1]	IMAC + FLAG [1]	not published
Omicron BA.4/5	CHO^{2353} stable pool [2]	non-affinity [3]	not published
Omicron BA.2.12.1	CHO^{55E1} transient [1]	IMAC + FLAG [1]	not published
Omicron XBB.1.5	CHO^{55E1} transient [1]	IMAC + Strep [1]	not published
Omicron B.1.1.529	CHO^{55E1} transient [1]	IMAC + Strep [1]	not published
Beta	CHO^{55E1} transient [1]	IMAC [4]	not published
Gamma	CHO^{2353} stable pool [2]	non-affinity [3]	not published
Alpha	CHO^{55E1} transient [1]	spike-affinity [5]	not published
Delta	CHO^{2353} stable pool [2]	non-affinity [3]	[3]
Kappa	CHO^{2353} stable pool [2]	spike-affinity [5]	not published
Ancestral	CHO^{2353} stable pool [2]	IMAC [4]	[6]
BANAL-20-52	CHO^{2353} stable pool [2]	IMAC + Strep [1]	[7]
RaTG13	CHO^{2353} stable pool [2]	IMAC + Strep [1]	not published
Pangolin-GX	CHO^{2353} stable pool [2]	IMAC + Strep [1]	[7]
ZXC21	CHO^{2353} stable pool [2]	IMAC + Strep [1]	not published
Longquan-140	CHO^{2353} stable pool [2]	IMAC + Strep [1]	not published
Bat WIV1	CHO^{2353} stable pool [2]	IMAC + Strep [1]	[7]
SHC014	CHO^{2353} stable pool [2]	IMAC + Strep [1]	[7]
Bat SARSL	CHO^{2353} stable pool [2]	IMAC + Strep [1]	[7]
Civet SARS-CoV	CHO^{2353} stable pool [2]	IMAC + Strep [1]	not published
SARS-CoV-1	CHO^{2353} stable pool [2]	IMAC + Strep [1]	[7]
Bat HKU9	CHO^{2353} stable pool [2]	IMAC + Strep [1]	not published
Hedgehog HKU31	CHO^{2353} stable pool [2]	IMAC + Strep [1]	not published
MERS	CHO^{55E1} transient [1]	IMAC + Strep [1]	not published
HKU1	commercial (https://www.sinobiological.com/recombinant-proteins/hcov-hku1-cov-spike-40606-v08b-b)		
OC43	CHO^{2353} stable pool [2]	IMAC + Strep [1]	not published
Porcine HKU15	CHO^{2353} stable pool [2]	IMAC + Strep [1]	not published
Bat 512	CHO^{2353} stable pool [2]	IMAC + Strep [1]	not published
Bat 229E	CHO^{2353} stable pool [2]	IMAC + Strep [1]	not published

References

1. Stuible, M. *et al.* Rapid, high-yield production of full-length SARS-CoV-2 spike ectodomain by transient gene expression in CHO cells. *J. Biotechnol.* **326**, 21–27 (2021).
2. Joubert, S. *et al.* A CHO stable pool production platform for rapid clinical development of trimeric SARS-CoV-2 spike subunit vaccine antigens. *Biotechnol. Bioeng.* **120**, 1746–1761 (2023).
3. Akache, B. *et al.* Immunogenicity of SARS-CoV-2 spike antigens derived from Beta & Delta variants of concern. *Npj Vaccines* **7**, 1–7 (2022).
4. Akache, B. *et al.* Immunogenic and efficacious SARS - CoV - 2 vaccine based on resistin - trimerized spike antigen SmT1 and SLA archaeosome adjuvant. *Sci. Rep.* 1–17 (2021) doi:10.1038/s41598-021-01363-7.
5. Stark, F. C. *et al.* Intranasal immunization with a proteosome-adjuvanted SARS-CoV-2 spike protein-based vaccine is immunogenic and efficacious in mice and hamsters. *Sci. Rep.* **12**, 9772 (2022).
6. Colwill, K. *et al.* A scalable serology solution for profiling humoral immune responses to SARS-CoV-2 infection and vaccination. *Clin. Transl. Immunol.* **11**, e1380 (2022).
7. Renner, T. M. *et al.* Reduced cross-protective potential of Omicron compared to ancestral SARS-CoV-2 spike vaccines against potentially zoonotic coronaviruses. *Npj Viruses* **2**, 1–8 (2024).
